# Taxonomic review of the Palaearctic species of the *Cheilosia caerulescens*-group (Diptera, Syrphidae)

**DOI:** 10.3897/zookeys.662.11267

**Published:** 2017-03-22

**Authors:** Gunilla Ståhls, Anatolij V. Barkalov

**Affiliations:** 1 Ståhls, G., Finnish Museum of Natural History, Zoology unit, P.O. Box 17, FI-00014 University of Helsinki, Finland; 2 Barkalov, A. V., Institute of Systematics and Ecology of Animals, Russian Academy of Sciences, Siberian Branch, 11 Frunze str., 630091 Novosibirsk, Russia

**Keywords:** Barcoding, *Cheilosia caerulescens*, mtDNA COI, taxonomy

## Abstract

The Palaearctic species of the *Cheilosia caerulescens* group (Diptera: Syrphidae) are revised in this work. The species group belongs to the genus Cheilosia subgenus Taeniocheilosia Oldenberg. One new species is described from north Caucasus, Cheilosia (Taeniocheilosia) circassica **sp. n.** *Cheilosia primulae* Hering is established as a junior synonym of *Cheilosia laeviventris* Loew. Four lectotype designations are made. The species of the *Cheilosia caerulescens* group are redescribed and illustrated, and a table of diagnostic characters and an identification key to species are provided. MtDNA COI barcodes were generated for several specimens of C. (T.) caerulescens Meigen and other Cheilosia (Taeniocheilosia) and *Cheilosia* s. str. taxa. Parsimony and maximum likelihood analyses did not place the morphologically similar *C. hercyniae* Loew in the *C. caerulescens* group but among other Cheilosia (Taeniocheilosia) taxa. The following eight taxa are included in the Cheilosia (T.) caerulescens group of species: *Cheilosia armeniaca* Stackelberg, 1960, *C. caerulescens caerulescens* (Meigen, 1822), *C. caerulescens calculosa* Skufjin, 1977, *C. circassica* sp. n., *C. herculana* Brădescu, 1982, *C. kerteszi* Szilády, 1938, *C. laeviventris* Loew, 1857, and *C. venosa* Loew, 1857.

## Introduction

*Cheilosia* Meigen, 1822 (Diptera, Syrphidae) is the largest Palaearctic hoverfly genus with nearly 300 species listed (Peck 1988). Thompson et al. (2010) reported 439 species of *Cheilosia* known worldwide, and Vujić et al. (2013), Ricarte et al. (2014), Barkalov and Ståhls (2015), Barkalov and Ichige (2016) added altogether six species, thus the present total number is 445. Barkalov (2002) classified the *Cheilosia* species into 13 subgenera, of which nine were new (*Cheilosia* Meigen, 1822; *Endoiasimyia* Bigot, 1882 (= *Sonanomyia* Shiraki, 1930); *Taeniochilosia* Oldenberg, 1916 (= *Nigrocheilosia* Shatalkin, 1975); *Hiatomyia* Shannon, 1922; *Neocheilosia* Barkalov, 1983; *Eucartosyrphus* Barkalov, 2002; *Floccocheila* Barkalov, 2002; *Pollinocheila* Barkalov, 2002; *Montanocheila* Barkalov, 2002; *Nephocheila* Barkalov, 2002; *Conicheila* Barkalov, 2002; *Convocheila* Barkalov, 2002 and *Rubrocheila* Barkalov, 2002. Barkalov (2007) synonymized subgenus Nephocheila Barkalov, 2002 with *Nephomyia* Matsumura, 1916.

The Palaearctic bare eyed and black legged species of the subgenus Taeniocheilosia Oldenburg (as *Nigrocheilosia* Shatalkin) were revised by Barkalov and Ståhls (1997). They included the species Cheilosia (Taeniocheilosia) laeviventris Loew and C. (T.) venosa Loew in their species key, as these species occasionally have almost completely black legs, thus agreeing with the other *Taeniocheilosia* taxa. The two aforementioned taxa belong to the C. (Taeniocheilosia) caerulescens Meigen group of species, identified by typically having both bi-coloured legs (yellow and black) and infuscated wing cross-veins, which discerns them from other sg. Taeniocheilosia taxa. The structure of male genitalia in the *caerulescens* group agrees with that of the subgenus Taeniocheilosia (see Barkalov and Ståhls 1997).

The aim of the present study is to revise the Palaearctic species, to define the members of the C. (Taeniocheilosia) caerulescens group and to redescribe them. We were especially interested in testing the phylogenetic placement of *Cheilosia hercyniae* Loew, 1857 based on molecular sequences. The taxon belongs to the subgenus. Taeniocheilosia and has bi-colored legs and very slightly infuscated wing crossveins (or with only yellowish wing veins without infuscation), thus partly agreeing with character states of other *Cheilosia caerulescens* group taxa.

In addition to the morphological studies of the C. (Taeniocheilosia) caerulescens group taxa, mtDNA COI barcodes were obtained for recently collected *C. caerulescens* specimens, and also for representative taxa of subgenera *Taeniocheilosia* (including C. (T.) hercyniae), *Eucartosyrphus* and *Cheilosia* s. str. These sequence data were analyzed using parsimony and maximum likelihood to explore the placements of the mentioned taxa.

## Materials and methods

### Morphological studies

In the material examined, the collections where the specimens are deposited are indicated between square brackets after each specimen. Type localities and holotype-holding institutions are specified for each species. Identification and location labels are indicated with single quotation marks. Handwritten information on labels is indicated.

Terminology follows Thompson (1999) and Barkalov and Cheng (2004) for most terms. Two of the most characteristic morphological features of *Cheilosia* taxa, the central area of the face below the antennae that is produced anteriorly, and the distinct lateral narrow area between the face and the eye, have been identified by different terms in various publications. The central area of the face below the antennae that is produced anteriorly is named facial knob by Barkalov and Ståhls (1997), central prominence by Nielsen and Claussen (2001), central knob by Barkalov and Cheng (2004), facial tubercle by Claussen and Ståhls (2007), to name a few. The lateral narrow area between the face and the eye (the elongated tentorial arms) are termed paraface (McAlpine 1981, Thompson 1999), ocular strips (Speight 1987), eye margin (Barkalov and Ståhls 1997, Barkalov and Cheng 2004), orbital stripes (Thompson 1999, Nielsen and Claussen 2001), facial stripes (Hippa and Ståhls 2005), or parafacialia (Claussen and Ståhls 2007). There is no confusion as to which morphological structures of the *Cheilosia* flies the different terms refer to, and no clear arguments have been presented for establishing a preference among them. In the present study we use ‘facial knob’ and ‘parafacia’.

The material comprising adult flies consists of dry, pinned specimens from the following museums, institutions and private collections:

**Coll. C. Claussen** Private collection of Claus Claussen, Germany

**Coll. D. Doczkal** Private collection of Dieter Doczkal, Germany

**Coll. T. Romig** Private collection of Tomas Romig, Austria

**ETH** Eidgenössische Technische Hochschule Zürich, Institut für Angewandte Entomologie, Switzerland

**FSUNS** Insect collection of the University of Novi Sad, Serbia

**ISEA** Institute of Systematics and Ecology of Animals (earlier BIN Biological Institute), Novosibirsk, Russia

**MGAB** Muséum d’Histoire Naturelle “Grigore Antipa”, Bucharest, Romania

**MNHN** Musée National d’Histoire Naturelle, Paris, France

**MZH** Finnish Museum of Natural History, University of Helsinki, Finland

**NMW** Naturhistorisches Museum, Wien, Austria

**RMNH** Naturalis Biodiversity Center [formerly Rijksmuseum van Natuurlijke Historie], Leiden, the Netherlands

**VSU** Voronezh State University, Russia

**ZIN** Zoological Institute of the Russian Academy of Sciences, St Petersburg, Russia

**ZMHU** Museum für Naturkunde – Leibniz-Institut für Evolutions- und Biodiversitätsforschung, Berlin, Germany [formerly MNB, Museum für Naturkunde der Humboldt-Universität]

Male genitalia were dissected, macerated in 10% KOH, soaked in water and neutralized with 10% acetic acid, and stored in glycerol in plastic microvials on the same pins as the specimens.

**Figure 1.**
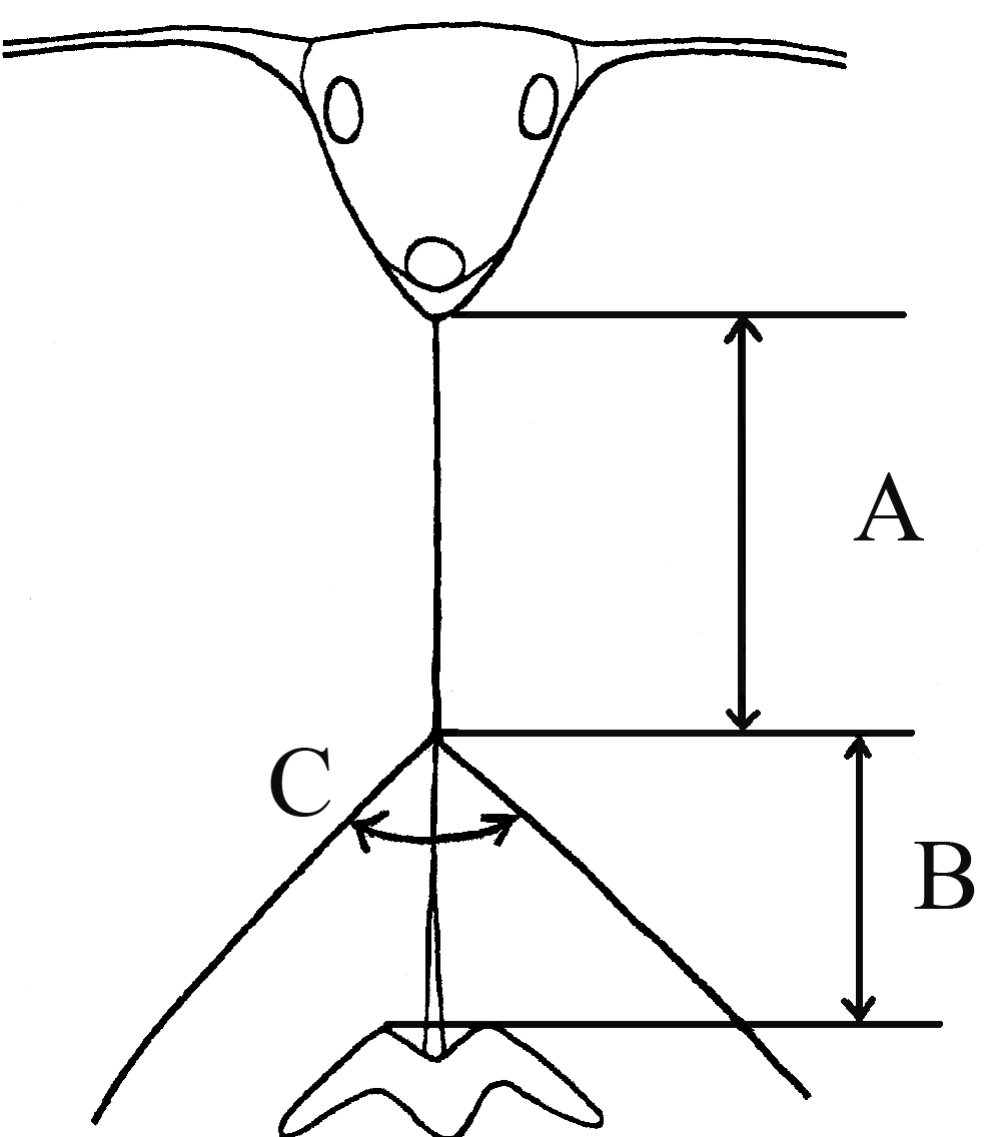
Head of male, dorsal view, comparison of lengths of eye-contiguity (**A**) and frons (**B**), and frontal angle (**C**).

For each species figures are provided for the attributes considered relevant for species recognition. Body length was measured dorsally from the anterior margin of lunula to the tip of the abdomen. Drawings are provided of the male genitalia, the profile of the face and the basoflagellomere for the males; and of the dorsal view of frons and basoflagellomere of the females. The figures of male heads show the distribution of pollinosity and pilosity, in females the distribution of pollinosity. The dorsal aspect of the male head is shown in Fig. 1. Photographs are provided to illustrate selected characters of *Cheilosia c. caerulescens*, *C. laeviventris* and *C. venosa*.

When necessary, a lectotype has been designated and labelled accordingly in order to fix the concept of the taxon in question and to ensure the universal and consistent interpretation of the same.

The distribution given in Peck (1988) is cited with additional data from Marcos García (1985), Brădescu (1991), Maibach et al. (1992), Vujić (1996, and unpublished), Barkalov and Ståhls (1997), Reemer et al. (2009) and Ball et al. (2011). In Peck (1988) and Vujić (1996) the country Yugoslavia is used; in the present paper we indicate in which of the presently existing Balkan countries the species are distributed according to the present political borders. New distributional range data (countries) resulting from the present study are indicated with an asterisk.

## Molecular studies

### Laboratory procedures

DNA was extracted from 1–3 legs of dry pinned or ethanol preserved specimens using the Nucleospin Tissue DNA extraction kit (Machery-Nagel, Düren, Germany) following the manufacturer’s protocols and then re-suspended in 50 µl of ultra-pure water.

The universally conserved primers LCO1490 and LCO2198 (Folmer et al. 1994) were used for amplifying and sequencing the 5’ end of mtDNA COI. PCR reactions were carried out using GE Ready-to-Go PCR beads in 25 µl reaction aliquots containing 1–2 µl DNA extract, 1 µl of each primer (at 10 pmol/µl) and ultrapure water. Thermocycler conditions were initial denaturing at 95°C 2 min, 29 cycles of 30 s denaturing at 94°C, 30 s annealing at 49°C, 2 min extension at 72°C, followed by a final extension of 8 min at 72°C. Amplified PCR products were visualized on 1.5% agarose gels and treated with Exo-SapIT (USB Affymetrix, Ohio, USA) prior to sequencing. Both PCR primers were used for sequencing. The Big Dye Terminator Cycle Sequencing Kit (version 3.1) (Applied Biosystems, Foster City, CA, USA) was used on an ABI 3730 (Applied Biosystems, Foster City, CA, USA) genetic analyzer at the Sequencing Service Laboratory of the Finnish Institute for Molecular Medicine (FIMM), University of Helsinki. The sequences were edited for base-calling errors and assembled using Sequencher™ (version 4.9) (Gene Codes Corporation, Ann Arbor, MI, USA). All new sequences were submitted to the European Nucleotide Archive database (www.ebi.ac.uk/ena) (see Table 2 for accession numbers).

**Table 2.** Molecular samples with EMBL ENA accession codes.

Labcode	Species	Locality	Sex	EMBL ENA accession
MZH_Y2067	*Cheilosia albipila* Meigen	RUSSIA, Caucasus, Krasnodar krai, Guamskoe canyon, 1700m, Kurdzips river area, 20.V.2015, E. Rättel, O. Gerzovsky leg.	Female	LT707495
MZH_Y1274	*Cheilosia albitarsis* (Meigen)	FINLAND, 66941:34004, N: Sibbo, Hindsby, 13.VI.2009, G. Ståhls leg.	Female	LT707496
MZH_Y2053	*Cheilosia caerulescens caerulescens* (Meigen)	SWITZERLAND, Valais, nr Simplon Dorf, 1650 m, 46°12'19"N, 8°02'25"E, 24.IV.2015, G. Ståhls leg.	Male	LT707497
MZH_Y2054	*Cheilosia caerulescens caerulescens* (Meigen)	SWITZERLAND, Valais, nr Simplon Dorf, 1650 m, 46°12'19"N, 8°02'25"E, 24.IV.2015, G. Ståhls leg.	Male	LT707498
MZH_Y2203	*Cheilosia caerulescens caerulescens* (Meigen)	SWITZERLAND, Valais, Rossboden, 46.196058N, 08.026283E, 25.V.2015, 2000m, G. Ståhls leg.	Male	LT707499
MZH_Y2205	*Cheilosia caerulescens caerulescens* (Meigen)	SWITZERLAND, Valais, Rossboden, 46.196058N, 08.026283E, 25.V.2015, 2000m, G. Ståhls leg.	Male	LT707500
MZH_Y2219	*Cheilosia caerulescens caerulescens* (Meigen)	SWITZERLAND, Valais, Rossboden, 46.196058N, 08.026283E, 25.V.2015, 2000m, G. Ståhls leg.	Male	LT707501
CNCDB434_11	*Cheilosia caerulescens caerulescens* (Meigen)	FRANCE, Haute, Montagne de Lure Jas de Bailles, 1300 m, C. Kassebeer leg.	Female	JN991966
MZH_Y2137	*Cheilosia derasa* Loew	SLOVENIA, Triglav NP, 46.303°N, 13.762°E, 22.VII.2012, 1520–1685m, S.M. Blank coll.	Female	LT707508
MZH_Y2063	*Cheilosia gagatea* Loew	SWITZERLAND, Valais, Rossboden, 46.196058N, 08.026283E, 25.V.2015, 2000m, G. Ståhls leg.	Male	LT707507
FSUNS_E86	*Cheilosia grisella* Becker	SWITZERLAND, Valais, nr Simplon Dorf, 1650 m, 46°12'19"N, 8°02'25"E, 7.VI.2013, A. Vujic leg.	Female	LT707510
MZH_G445	*Cheilosia hercyniae* Loew	MONTENEGRO, Durmitor, 1997, A. Vujic leg.	Female	AY533355
MZH_Y2048	*Cheilosia impudens* Becker	SWITZERLAND, Valais, nr Simplon Dorf, 1650 m, 46°12'19"N, 8°02'25"E, 24.IV.2015, G. Ståhls leg.	Male	LT707502
FSUNS_E87	*Cheilosia insignis* Loew	SERBIA, Tara, Brusnica, 27-IV.2012, Vujić A., Radenković, S., Likov, L. leg.	Female	LT707512
MZH_G367	*Cheilosia kerteszi* (Szilady)	MONTENEGRO, Durmitor, 1997, A. Vujic leg.	Male	AY533344
MZH_Y2045	*Cheilosia laeviseta* Claussen	SWITZERLAND, Valais, Rossboden, 46.196058N, 08.026283E, 25.V.2015, 2000m, G. Ståhls leg.	Male	LT707505
MZH_Y2099	*Chelosia longula* (Zetterstedt)	FINLAND, 69745:35305, Sb: Kuopio, Kolmisoppi, 19.VIII.2015, G. Ståhls & E. Rättel leg.	Female	LT707514
MZH_jka 05–00793	*Cheilosia nigripes* (Meigen)	FINLAND, Kesälahti, Pivanka, 12.VI.2005, J. Kahanpää leg.	Female	BOLD process ID FIDIP2504–12
FSUNS_E83	*Cheilosia nivalis* Becker	SWITZERLAND, Valais, nr Simplon Dorf, 1650 m, 46°12'19"N, 8°02'25"E, 7.VI.2013, A. Vujic leg.	Male	LT707509
MZH_Y2061	*Cheilosia pedemontana* Rondani	SWITZERLAND, Valais, Rossboden, 46.196058N, 08.026283E, 25.V.2015, 2000m, G. Ståhls leg.	Male	LT707504
FSUNS_E84	*Cheilosia personata* Loew	SWITZERLAND, Valais, nr Simplon Dorf, 1650 m, 46°12'19"N, 8°02'25"E, 7.VI.2013, A. Vujic leg.	Male	LT707511
MZH_Y2059	*Cheilosia pubera* (Zetterstedt)	FINLAND, Rantasalmi 30.V.2015, E. Rättel leg.	Female	LT707506
MZH_Y1874	*Cheilosia sibirica* Becker	RUSSIA, Altay, Teletskoe lake area, 51°47'29.502N, 87°19'06.11E, 23–25.VI.2013, G. Ståhls leg.	Female	LT707513
MZH_Y2066	*Cheilosia schnabli* Becker	RUSSIA, Caucasus, Krasnodar krai, near Apscheronsk, 700m, 20.V.2015, E. Rättel leg.	Male	LT707517
MZH_Y1960	*Cheilosia scutellata* (Fallén)	SPAIN, Grazalema NP, 36°43'03"N, 5°20'06"W, nr puerto Los Alamillos, 800m, 14.VI.2014, G. Ståhls leg.	Male	LT707516
MZH_Y1962	*Cheilosia soror* (Zetterstedt)	GREECE, Samos island, 37°47.313N, 26°49.412E, 173m, nr Manolates, 14.V.2010, G. Ståhls leg.	Male	LT707515
MZH_Y2049	*Cheilosia vicina* (Zetterstedt)	SWITZERLAND, Valais, nr Simplon Dorf, 1650 m, 46°12'19"N, 8°02'25"E, 24.IV.2015, G. Ståhls leg.	Male	LT707503
MZH_Y1657	Pelecocera (Chamaesyrphus) scaevoides (Fallén)	FINLAND, N: Raseborg, Ekenäs, 14.VIII.2012, G. Ståhls & E. Rättel leg.	Female	AY533320
MZH_Y1973	*Ferdinandea ruficornis* (Fabricius)	RUSSIA, Primorsk region, Sikhote-Alin reserve, nr Khanov camp, 44°53'72"N, 136°20'13"E, 1.IX.2014, G. Ståhls & E. Rättel leg.	Female	LT707518

### Sequence analyses

Full-length mtDNA COI barcodes were generated for 25 *Cheilosia* specimens. We additionally included shorter barcodes (450 bp) of the species *Cheilosia kerteszi* and *C. hercyniae*, and one barcode of *C. caerulescens* was retrieved from the Barcoding of Life Database (Table 2). Two outgroup species of family Syrphidae, tribe Rhingiini were used: Pelecocera (Chamaesyrphus) scaevoides (Fallén, 1817) and *Ferdinandea ruficornis* (Fabricius, 1775) (Table 2). All analyses were rooted on Pelecocera (C.) scaevoides.

Parsimony analyses for all sequence datasets were performed in NONA (Goloboff, 1999) spawn with the aid of WINCLADA (Nixon, 2002) using the heuristics search algorithm with 1000 random addition replicates (mult*1000), holding 100 trees per round (hold/100), maxtrees set to 100 000 and applying tree-bisection–reconnection branch swapping. Nodal support for each tree was assessed using non-parametric bootstrap resampling with 1000 replicates using WINCLADA. Maximum Likelihood (ML) analysis was done in MEGA version 6 using the General Time Reversible (GTR) model with gamma distributed rates among sites based on model-testing results as implemented in the software (Tamura et al. 2013). Branch support with 1000 bootstrap replications, and pairwise uncorrected *p*-distances were also calculated using MEGA.

## Results

### Taxonomy

**Figure 2.**
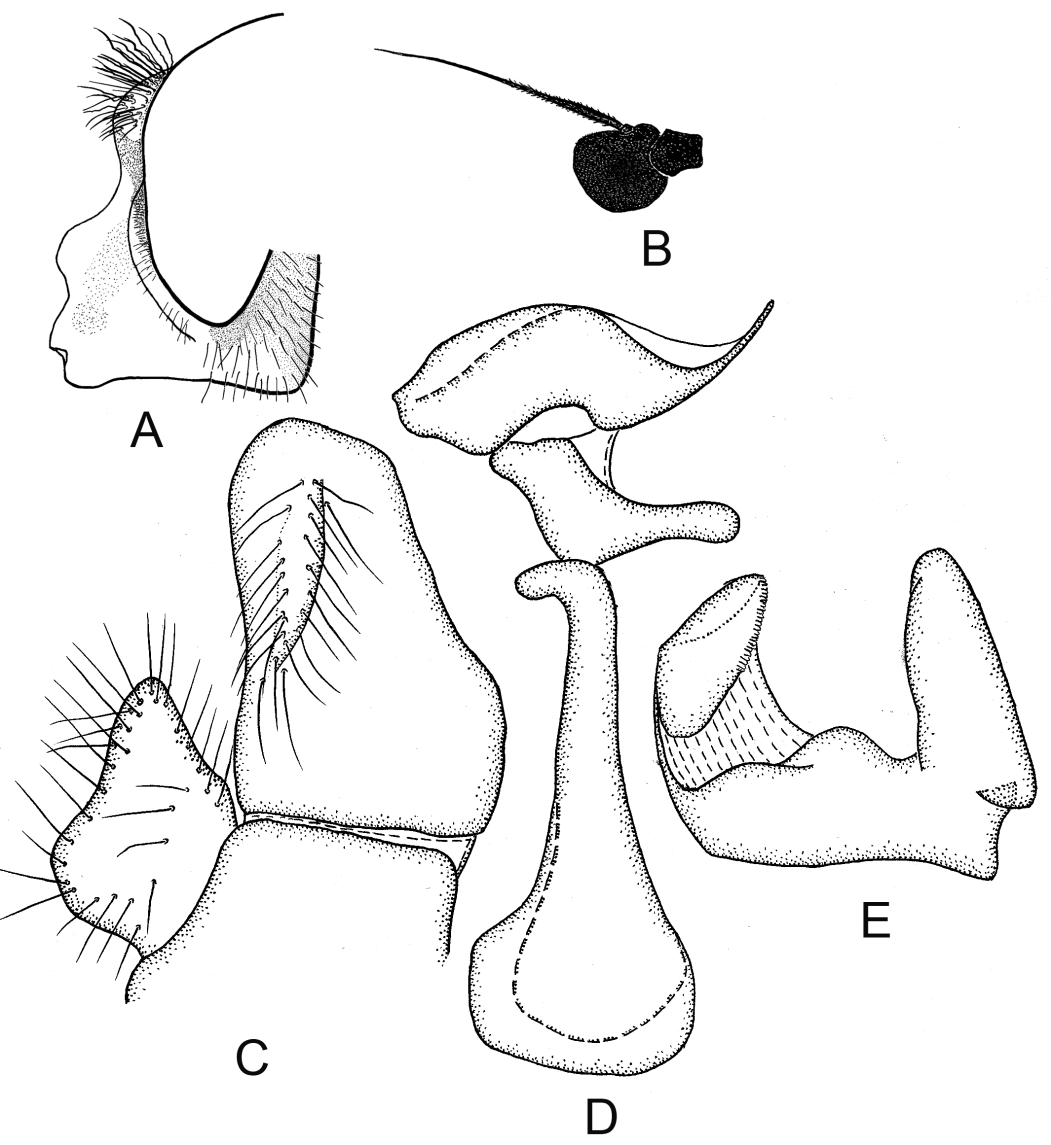
*Cheilosia armeniaca* Stackelberg, male. **A** Head, lateral view **B** Antenna, lateral view **C–E** male genitalia, lateral, from left to right: **C** Cercus+surstylus **D** Aedeagal complex **E** Superior lobe.

**Figure 3.**
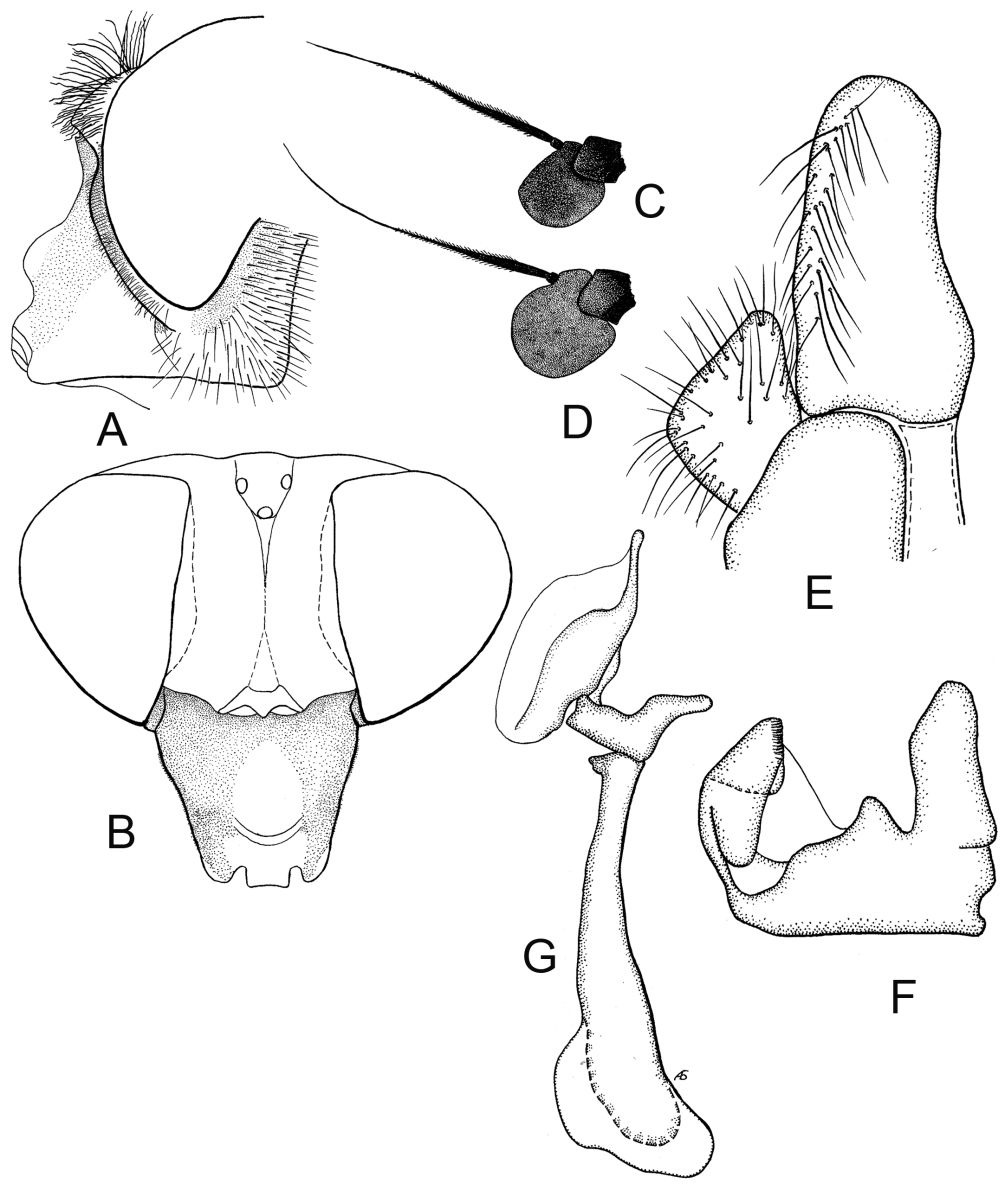
*Cheilosia caerulescens caerulescens* (Meigen). **A** Head of male, lateral view **B** Head of female dorsal view **C** Antenna of male, lateral view **D** Antenna of female, lateral view **E–G** male genitalia, lateral, from left to right: **E** Cercus+surstylus **F** Superior lobe **G** Aedeagal complex.

**Figure 4.**
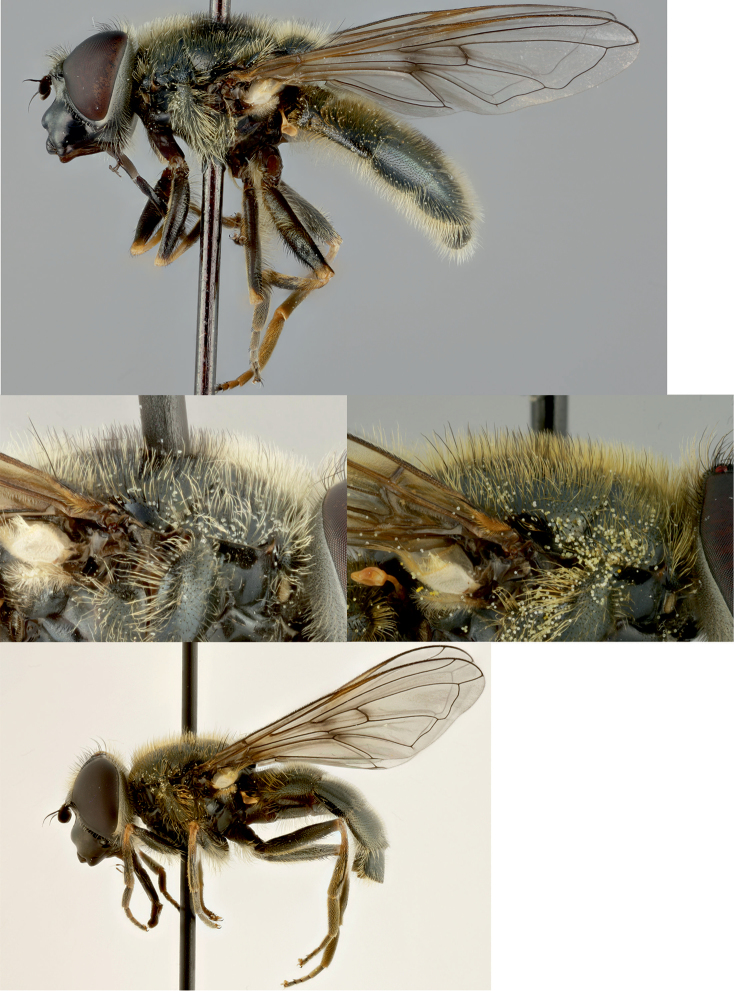
*Cheilosia caerulescens caerulescens* (Meigen). **A** Male, lateral view **B, C** Thorax of male lateral view, variability of composition of pilosity on scutum **D** Female, lateral view.

**Figure 5.**
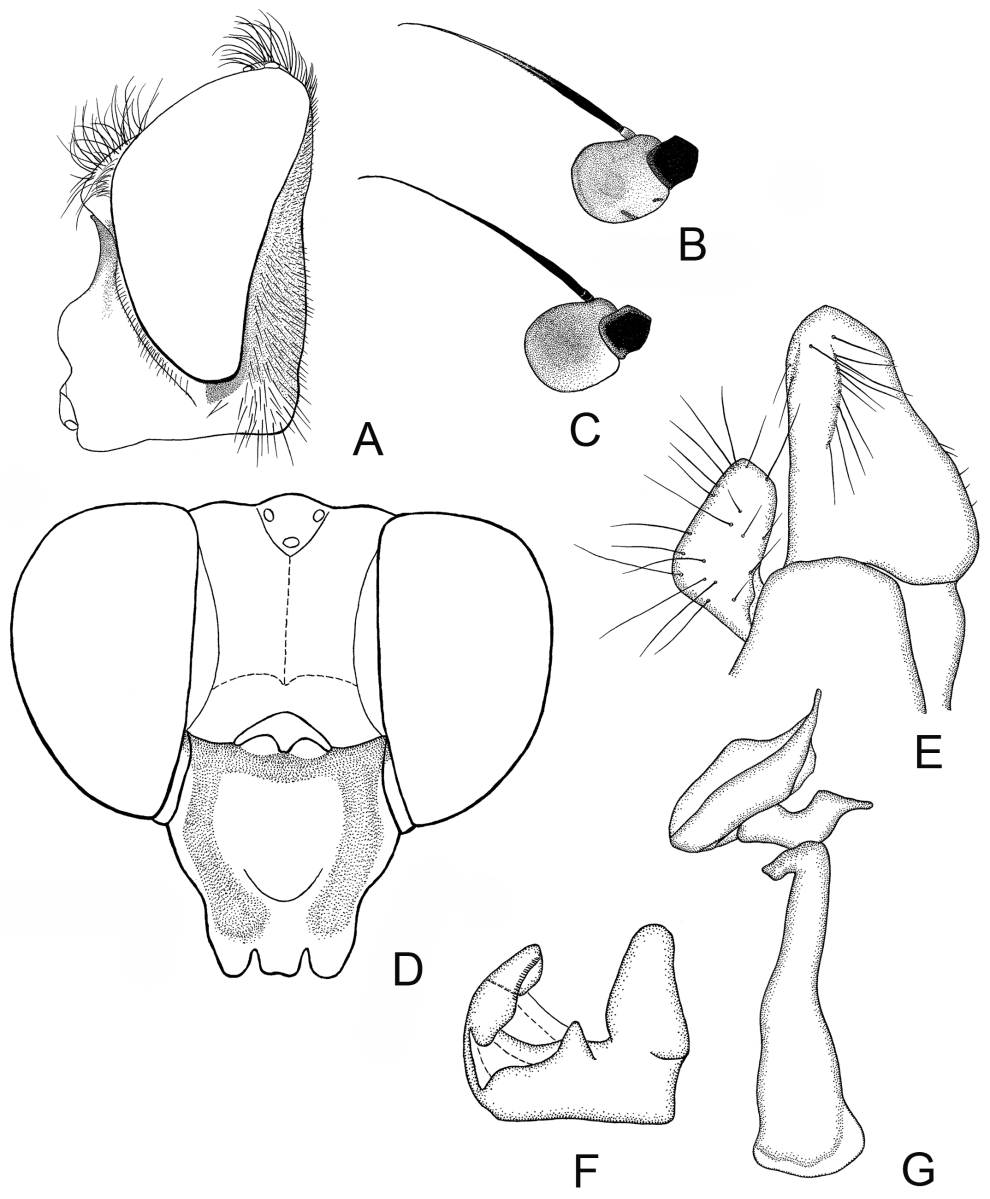
*Cheilosia caerulescens calculosa* Skufjin, male. **A** Head of male, lateral view **B** Antenna of male lateral view **C** Antenna of female lateral view **D** Head of female dorsal view **E–G** male genitalia, lateral, from left to right: **E** Cercus+surstylus **F** Superior lobe **G** Aedeagal complex

**Figure 6.**
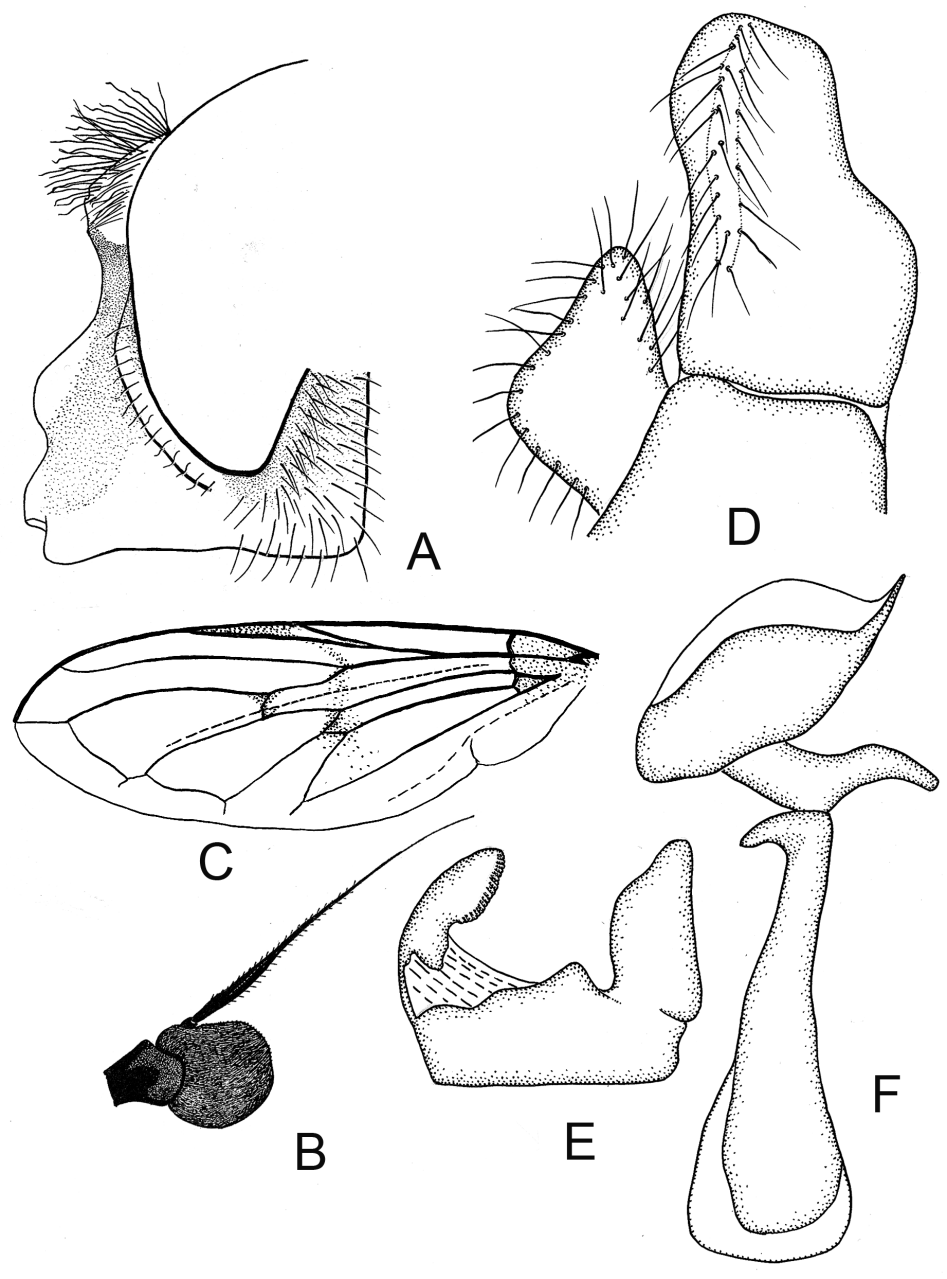
*Cheilosia circassica* sp. n. male. **A** Head, lateral view **B** Antenna, lateral view **C** Wing **D–F** male genitalia, lateral, from left to right: **D** Aedeagal complex **E** Superior lobe **F** Cercus+surstylus.

**Figure 7.**
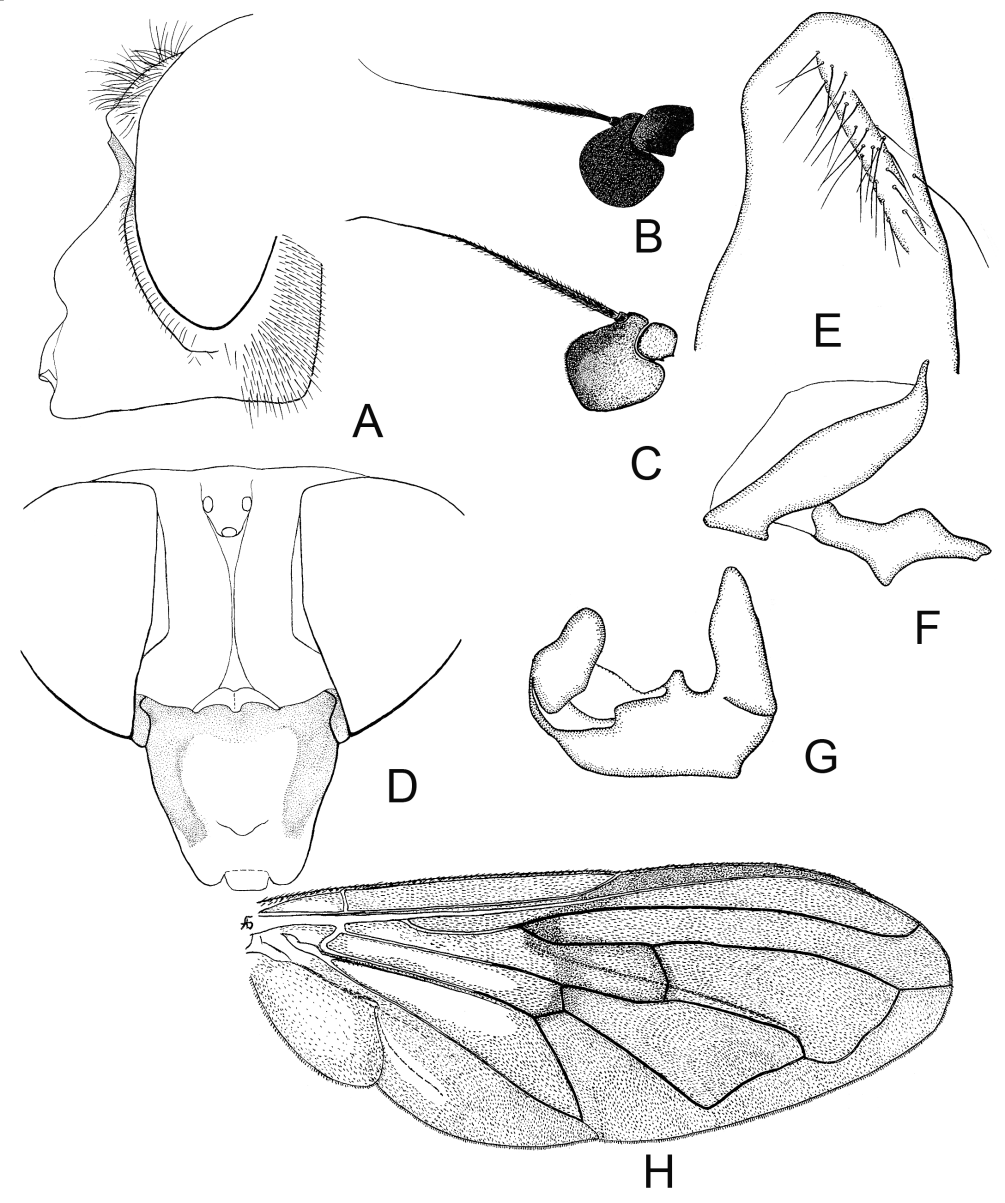
*Cheilosia herculana* Brădescu. **A** Male head, lateral view **B** Antenna of male lateral view **C** Antenna of female, lateral view **D** Head of female, dorsal view **E–G** male genitalia, lateral, from left to right: **E** Surstylus (dorsal part) **F** Aedeagus **G** Superior lobe **H** Male wing.

**Figure 8.**
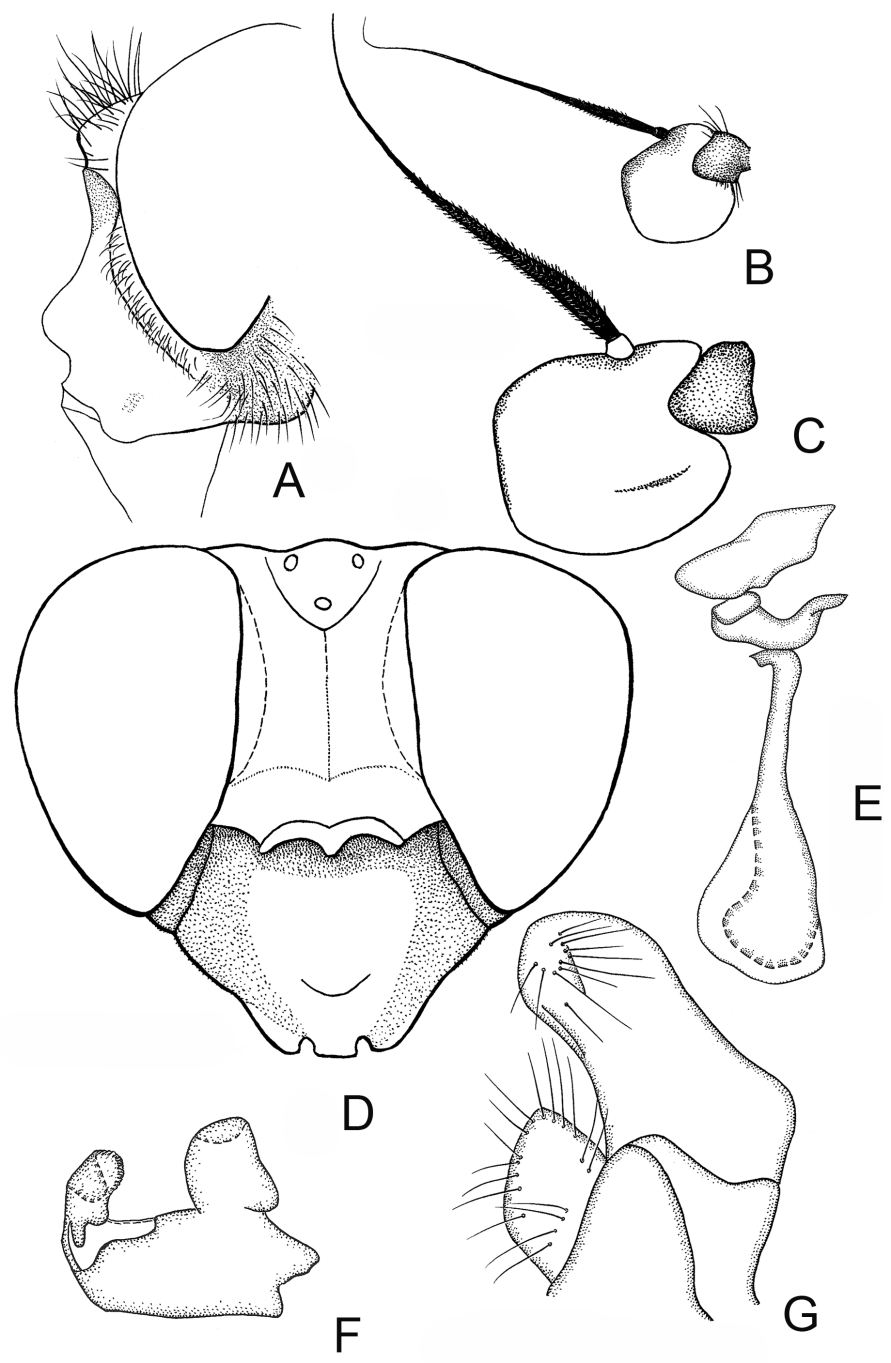
*Cheilosia hercyniae* Loew. **A** Male head, lateral view **B** Antenna of male, lateral view **C** Antenna of female, lateral view **D** Head of female, dorsal view **E–G** male genitalia, lateral, from left to right: **E** Aedeagal complex **F** Superior lobe **G** Cercus+surstylus.

**Figure 9.**
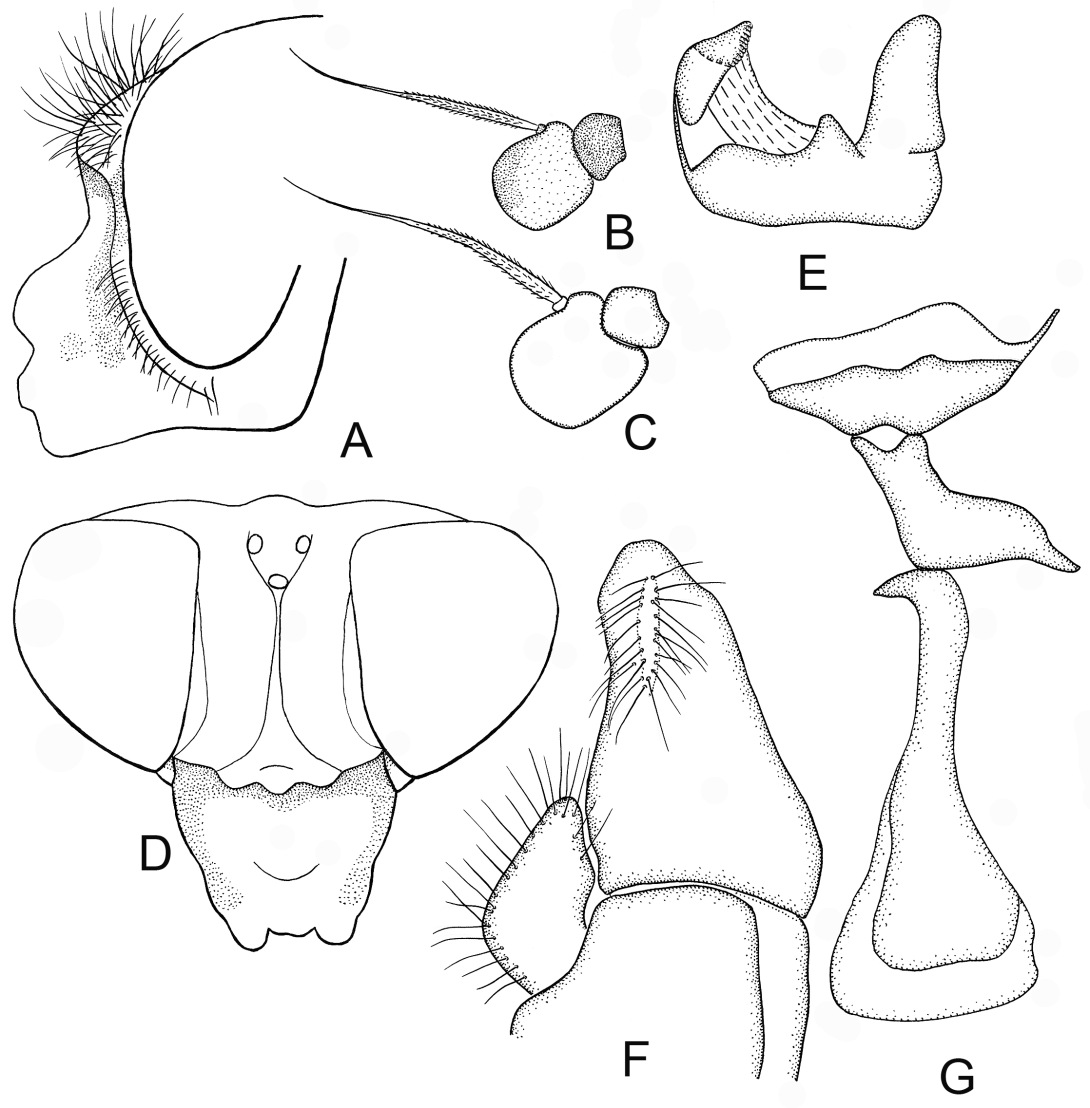
*Cheilosia kerteszi* Szilády. **A** Male head, lateral view **B** Antenna of male, lateral view **C** Antenna of female, lateral view **D** Head of female, dorsal view **E–G** male genitalia, lateral, from left to right: **E** Superior lobe **F** Cercus+surstylus **G** Aedeagal complex.

**Figure 10.**
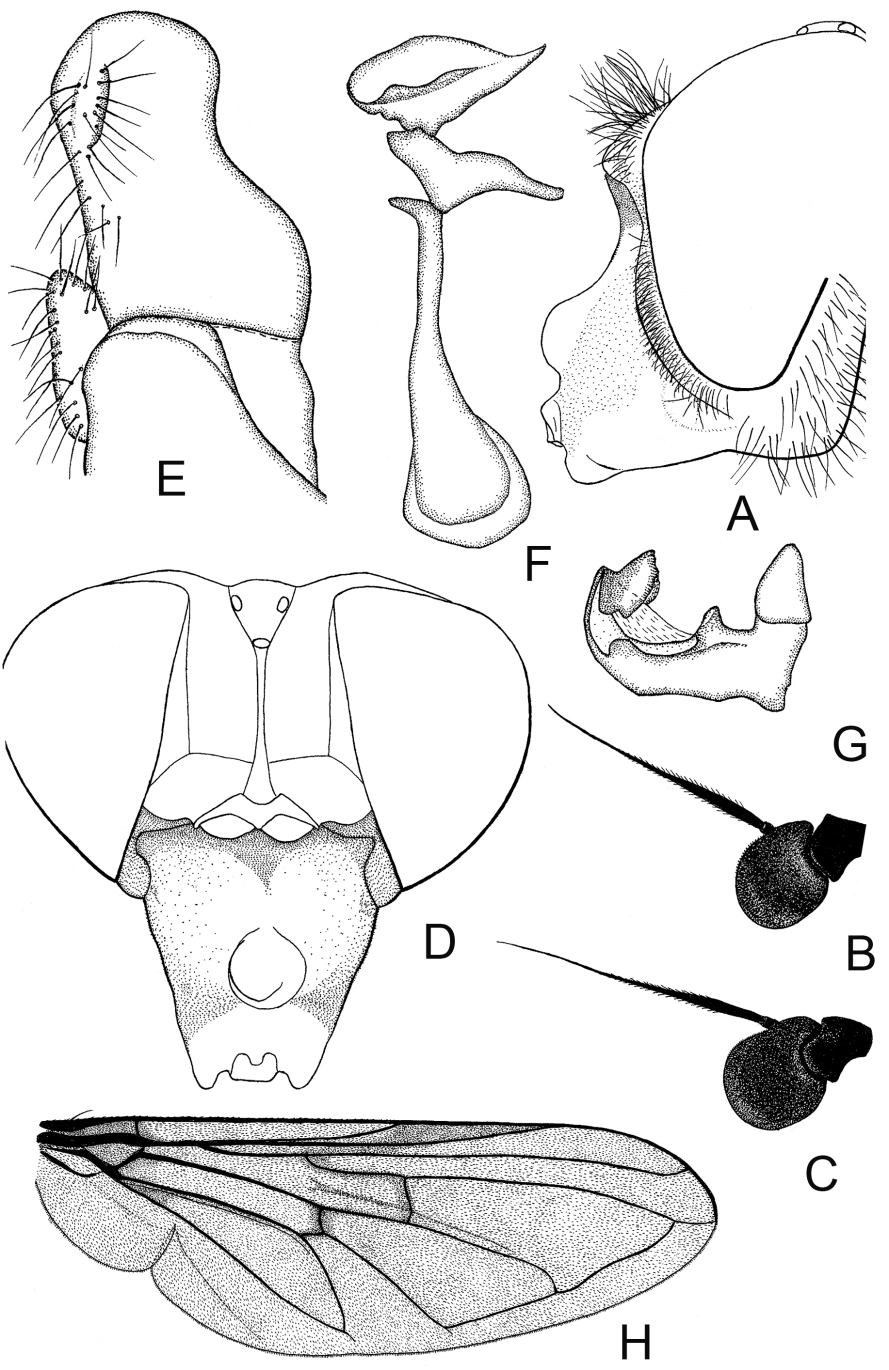
*Cheilosia laeviventris* Loew. **A** Male head, lateral view; B antenna of male, lateral view **C** Antenna of female, lateral view **D** Female head, dorsal view **E–G** male genitalia, lateral, from left to right: **E** Cercus +surstylus **F** Aedeagal complex **G** Superior lobe **H** Male, wing.

**Figure 11.**
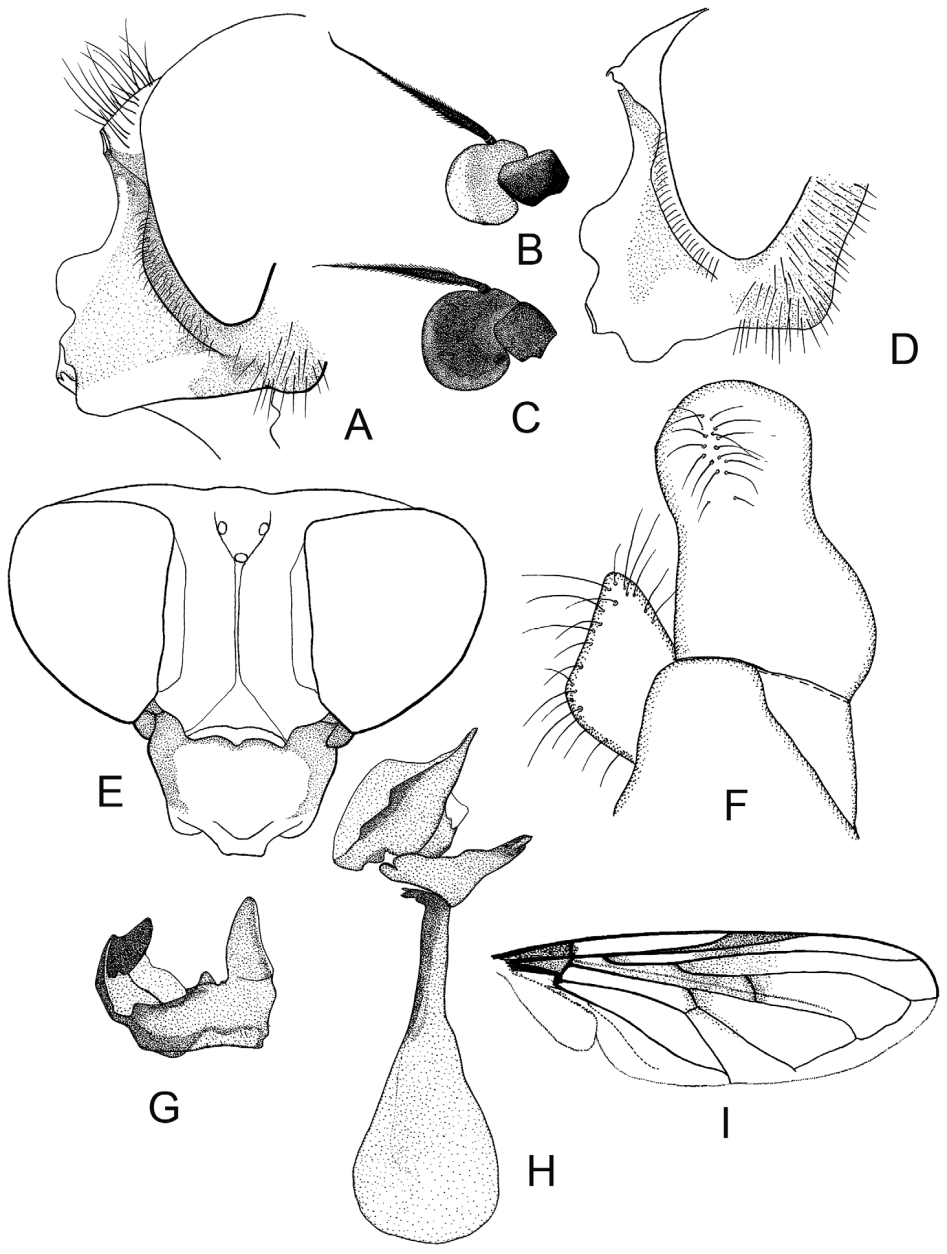
*Cheilosia venosa* Loew. **A** Male head, lateral view **B** Antenna of male, lateral view **C** Antenna of female, lateral view **D** Female head, lateral view **E** Female head dorsal view **F–H** male genitalia, lateral, from left to right: **F** Cercus+surstylus **G** Superior lobe **H** Aedeagal complex **I** Male, wing.

**Figure 12.**
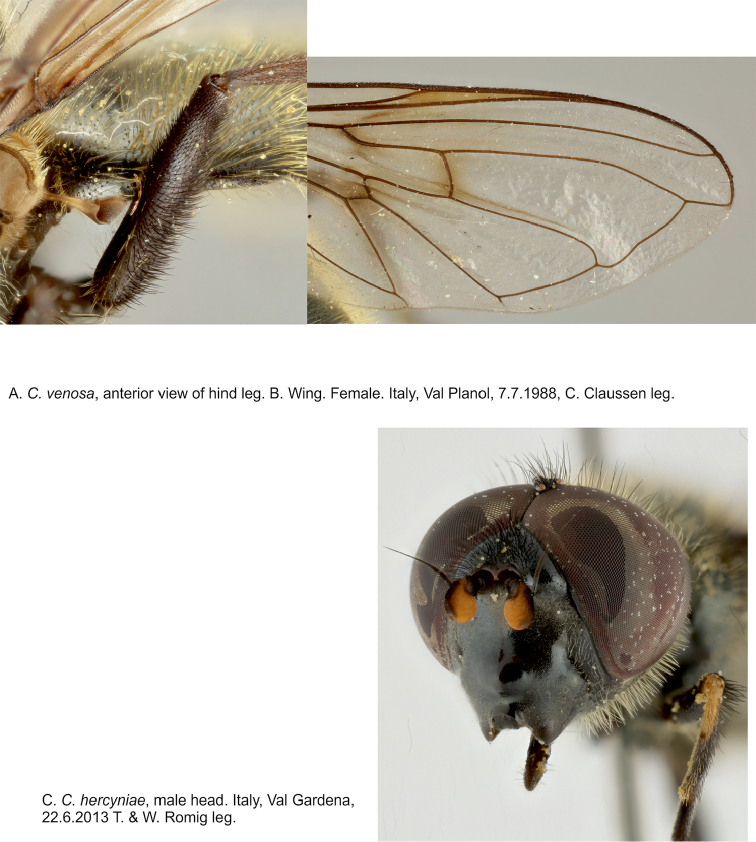
**A, B** *Cheilosia venosa*, metaleg and wing **C** *C. hercyniae*, male head.

The Cheilosia (Taeniocheilosia) caerulescens group of species can be distinguished from all other *Cheilosia* by the combination of the following characters: eye bare, distinctly darkened cross veins of wing, comparatively broad body, usually bi-colored legs, and characteristics of male genitalia structures: superior lobes with distinct left and right process, apical sclerite of aedeagus elongated in lateral direction (Figs 2–12). The following taxa are included in the Cheilosia (T.) caerulescens group of species: *Cheilosia armeniaca* Stackelberg, 1960, *C. caerulescens caerulescens* (Meigen, 1822), *C. caerulescens calculosa* Skufjin, 1977, *C. circassica* sp. n., *C. herculana* Brădescu, 1982, *C. kerteszi* Szilády, 1938, *C. laeviventris* Loew, 1857, and *C. venosa* Loew, 1857. Diagnostic characters separating the species and subspecies of the *caerulescens* group and *Cheilosia hercyniae* are summarized in Table 1.

**Table 1.** Diagnostic characters of species of the *Cheilosia caerulescens* group and *Cheilosia hercyniae*.

	*armeniaca*	*c. caerulesc*	*c. calculosa*	*herculana*	*hercyniae*	*kerteszi*	*laeviventris*	*teberda*	*venosa*
Antennal pits	confluent	confluent	separated	confluent	separated	separated	≈separated	confluent	confluent
Arista	pilose	pilose	bare	pilose	pilose	pilose	pilose	pilose	pilose
Pilosity on vertical triangle	whitish	black	black	black and some pale	black	some pale	black	white	black
Black bristles on post-alar callus	absent	present	present	absent	present	present	present	absent	present
Hair patches on katepisternum	connected	connected	connected	connected	widely divided	connected	divided	connected	narrowly connected
Distribution of microtrichia on wing	with some bare areas	uniformly microtrichose	uniformly microtrichose	with some bare areas	uniformly microtrichose	uniformly microtrichose	uniformly microtrichose	uniformly microtrichose	uniformly microtrichose
R4+5 of wing	straight	slightly curved	straight	straight	straight	straight	straight	straight	curved
Mesofemur: apico-posterior area	pale pile	pale pile, few black pile	black pile	pale pile	pale pile, few black pile	pale pile	black pile	pale pile	black pile
Metafemur: antero-basal area	long pale pile	long pale pile	long pale pile	long pale pile	pale pile	long pale pile	short black pile	long pale pile	black pile of different lengths

### Key to species of Cheilosia (T.) caerulescens group and C. (T.) hercyniae

**Table:** 

1	Cell bm and cell cup of wing with distinct areas bare of microtrichia (Fig. 7H)	**2**
–	Cell bm of wing completely microtrichose (Fig. 10H)	**3**
2	Face with dense pollinosity, frons with yellow pile mixed with some black pile, hypopygium Fig. 7E–G ***herculana* Brădescu, 1982**
–	Face with very slight pollinosity, frons with only pale pile, hypopygium Fig. 2C–E	***armeniaca* Stackelberg, 1960**
3	Apical half of posterior surface of mesofemur and basal part of anterior surface of metafemur with long black pile	**4**
–	Apical half of posterior surface of mesofemur and basal part of anterior surface of metafemur with long light pile (but metafemur only apico-ventrally sometimes with a few black pile)	**5**
4	Hind margin of scutellum without black bristles or bristle-like pile. R_4 + 5_ of wing distinctly curved (Fig. 11I; 12B); face with lower part not very prominent (Fig. 11A and D). Hypopygium Fig. 11F–H	***venosa* Loew, 1857**
–	Hind margin of scutellum with black bristles or bristle-like pile. R_4 + 5_ of wing not curved (Fig. 10H); face with lower part prominent (Fig. 10A, D). Hypopygium Fig. 10E–G	***laeviventris* Loew, 1857**
5	Basoflagellomere of male with distinct anterodorsal angle, completely bright orange or orange with darkened dorsal corner (Figs 8B, 12C), basoflagellomere of female big, bright orange (Fig. 8C). Hypopygium Fig. 8E–G	***hercyniae* Loew, 1857**
–	Basoflagellomere of male without anterodorsal angle, reddish brown to black (Figs 9B, 6B), basoflagellomere of female reddish brown to black, smaller (Fig. 9C)	**6**
6	Abdominal tergites with erect yellow pile only	**7**
–	Abdominal tergites with erect to semi-erect yellow pile laterally, medially at least on tergite III with some black pile	**8**
7	Antennal pits separated, vertical triangle with black and a few yellow pile, scutum with yellow and black pile, postalar callus with few black bristles. Hypopygium as on Fig. 9E–G	***kerteszi* Szilády, 1938**
–	Antennal pits confluent, all pile on body white, postalar callus without any bristles Hypopygium Fig. 6D–F	***circassica* sp. n.**
8	Basal 1–3 segments of pro- and metatarsi dorsally yellow, arista appearing bare (Fig. 5B, C). Hypopygium Fig. 5E–G. Distribution: Russia, Lipetzk Region	***caerulescenscalculosa* Skufjin, 1977**
–	Basal 1–3 segments of pro- and metatarsi dorsally black or dark-brown, arista with short pile (Fig. 3C, D). Hypopygium Fig. 3E–G. Distribution: Widely distributed in Europe	***caerulescenscaerulescens* (Meigen, 1822)**

### Species descriptions

#### Cheilosia armeniaca

Taxon classificationAnimaliaDipteraSyrphidae

Stackelberg

Fig. 2

Cheilosia armeniaca Stackelberg, 1960: 439.
Cheilosia armeniaca : Stackelberg and Richter 1968: 245; Barkalov 1993: 712.

##### Type locality.

East slope of Mountain Kapudschikh, Armenia.

##### Type material studied.

Holotype, ♂, pinned, in ZIN. The holotype is labelled: ‘Vostochnyi skl. g. Kapudschikh, Armenia, V. Zaitzev, 17.VIII.1959’ [East slope of mtn. Kapudschikh, Armenia]; ‘Holotypus *Cheilosia armeniaca* Stackelberg’.

##### Description.

♂: Face in frontal view slightly divergent from level of antennal insertion to lower mouth edge, covered with very weak narrow strip of pollinosity (Fig. 2A). Facial knob rounded, not very protruding, shining. Parafacia pollinose in upper part and shining in lower part, in its broadest part nearly 1/2 the width (in its narrowest part about 1/3) of basoflagellomere. Frons slightly swollen, shining, with dense white wavy pile. Frontal angle ≈ 90° (Fig. 1, indicated with letter C). Lunula dark-brown. Antennal pits confluent. Vertical triangle with white pile. Eye-contiguity shorter than length of frons without lunula (A < B, see Fig. 1). Antenna dark-brown, basoflagellomere rounded (Fig. 2B). Arista with very short pile. Scutum, scutellum and pleura shining, with only very pale yellowish (almost whitish) pile, longer pile on pleura and posterior part of scutum distally wavy. Scutum and scutellum completely without black bristles or bristle-like pile. Postalar callus without black bristles. Anepisternum and katepisternum shining, with slight pollinosity, with white pile. Katepisternum with dorsal and ventral hair patches connected. Legs mainly black, knees narrowly yellow, tibiae also narrowly yellow at the extreme tip, 1–3 segments of meso- and 2–3 segments of metatarsi reddish (in the paratype metatarsi black with reddish tips of tarsomeres). Apical half of posterior surface of mesofemur with long light pile. Metafemur with short and long white pile ventrally. Halter yellow. Wing with all cross-veins darkened. Cell bm and cell cup of wing with areas bare of microtrichia. R_4 + 5_ of wing not distinctly curved. Abdomen oval, shining, densely punctated. Pile erect and white, longer on the lateral parts and shorter on the central parts of tergites. Sternite I with pollinosity, sternites II–IV shining. Pile erect, white on sternites. Hypopygium as on Fig. 2C–E.

*Size*. Body length 8.5 mm.

##### Additional material studied.

Georgia 1♂; ‘Georgia, Gornaia Tushetia, Omalo village 30.VII.1959 (Zaitsev)’ [ZIN].

##### Distribution.

Caucasus: Armenia, Georgia*.

##### Remark.

Female unknown.

#### Cheilosia caerulescens caerulescens

Taxon classificationAnimaliaDipteraSyrphidae

(Meigen)

Figs 3 , 4

Syrphus caerulescens Meigen, 1822: 295.
Chilosia coerulescens : Becker 1894: 375, Sack 1932: 65; Šuster 1959: 74; Séguy 1961: 36.
Cheilosia caerulescens : van der Goot 1981: 159; Marcos García 1985: 517; Čepelák 1986: 52; Šimić1987: 38; Brădescu 1991: 39; Stuke 2000: 22; Reemer et al. 2009: 147; Ball et al. 2011: 44.
Nigrocheilosia caerulescens : Vujić 1996:53.

##### Type locality.

Österreich [Austria].

##### Type material studied.

Lectotype, ♂, in MNHN. The original description is for both sexes. Becker (1902: 354) mentions only one male specimen which agrees with the original description and he designates it ‘Die Type’. The lectotype bears the following labels: ‘Meigen’ [handwritten in ink, round greyish collection label]; ‘caerulescens’ [Meigen’s handwriting].

##### Description.

Male: Fig. 4A. Face in anterior view slightly divergent from level of antennal insertion to lower mouth edge, with lower part protruding, with pollinosity laterally and below antenna (Fig. 3A). Facial knob moderate in size, shining. Parafacia rather narrow, in width slightly more than 1/2 the width of basoflagellomere, shining in lower part and with pollinosity on upper part narrowly along the eye. Frons slightly swollen, shining, with white and posteriorly some black pile, yellow pile anteriorly along eye inclined. Frontal angle about 90°. Lunula dark brown. Antennal pits confluent. Vertical triangle with black pile. Eye-contiguity about equal or slightly shorter than the length of frons without lunula. Scape and pedicel dark-brown, basoflagellomere small, dark brown to yellowish brown. Arista rather long, with short pile (Fig. 3C). Scutum shining (with bluish tinge), rather coarsely punctated, with whitish to yellowish erect, rather long pile of about even length, intermixed with black pile of same length or slightly longer. Scutellum with yellowish erect pile of same length as on scutum, margin with some longer black or yellow bristles. Postalar callus with 3–5 black bristles. Anepisternum and katepisternum shining, with very slight pollinosity, and with white long pile. Katepisternum with dorsal and ventral hair patches connected. Wing completely microtrichose, hyaline, crossveins darkened. R_4 + 5_ of wing not distinctly curved, angle of R_4 + 5_ and M_1_ about 90°. Halter yellow. Legs with distal part of femora and tibiae, and basal 1/3–1/2 of tibiae yellowish, tarsi dorsally dark-brown. Mesofemur posteriorly with white pile, metafemur anteroventrally and dorsally with long white pile, and ventrally with shorter (stronger) black pile. Abdomen slightly oval, shining (bluish tinge), with coarse punctation, with long erect yellow pile on tergite I, tergites II–IV with erect yellow pile laterally (longest on tergite II), and somewhat shorter erect yellow and black pile intermixed medially. Yellow pilosity on postero-lateral corners tergites II–IV inclined. Sternite I with pollinosity, sternites II–IV shining, all with long yellowish pile. Hypopygium as in Fig. 3E–G.

*Female*: Fig. 4D. Face as in the male. Eye-margin width about 1/2 the width of basoflagellomere. Frons coarsely punctuated, with whitish erect pile anteriorly, and with black erect pile on posterior part (Fig. 3B). Whitish pile on anterior part of frons inclined along eye. Vertical triangle with black and white pile. Basoflagellomere rounded, reddish brown to dark-brown (Fig. 3D). Scutum shining, with white pile of even length, with few scattered black pile of about the same length or slightly longer intermixed. Postalar callus with 0–3 black or yellow bristles. Scutellum margin with 2–4 black or yellow bristly-like pile. Metafemur with only yellow pile. Tibiae in basal 1/3 yellow, apically narrowly and obscurely yellow, with broad dark ring, tarsi dorsally brown or yellow, with brown segments 4–5 of protarsi, segment 5 of mesotarsi and segments 3–5 of metatarsi. Abdomen oval. All sternites shining. Otherwise as the male.

*Size*. Body length 6.5–12 mm.

##### Additional material studied.

Switzerland 2 ♂, 2 ♀ Zermatt, 1 600–1 700 m, 30.VII.1964 (v.d. Goot; Lucas) [RMNH]; 1 ♂ Zermatt, 30.7.1930 [MNB]; **Italy** 1 ♂, 1 ♀ Italia, Südtirol, W Tubre di Avigna 1 300–1 800 m 6.VII.1988 (Claussen) [ISEA], 1 ♂ Stilfserjoch [MNB], 2 ♂ Italia, Alpen, Vinschgau Martelltal, Lyfi-Alm, 2 000–2 500 m, 1.7.1995 (Doczkal) [coll. Doczkal], 1 ♂ Italy, Stelvio Park, Trafoi, 1 900–2 000 m, 20.6.1993 (Ståhls) [MZH]; **France** 1 ♂ ‘Le Lautaret, 4.VIII.26’ [MNHN], 1 ♀ ‘Le Lautaret, 2.VIII.26’ [MNHN], 1 ♂ ‘Le Lautaret, 12.VI.1919’ [MNHN]; **Austria** ‘Wien, Egger’ [MNB]. See also Table 2 for data molecular specimen vouchers also used for morphological study.

##### Distribution.

Austria, Belgium, Czech Republic, France, Germany, Great Britain, Italy, Netherlands, Montenegro, Poland, Romania, Spain, Slovakia, Switzerland.

##### Remarks.

Color of pile on scutum varies from almost yellow with some black pile to yellow with broad stripe of black pile between wing bases (Fig. 4B–C). Color of tarsi varies from dark to mainly yellow (especially in female). Some studied females do not have yellow bristles or bristle-like pile on scutellum hind margin. The color of basoflagellomere varies from dark brown to yellowish brown. We treat the taxon as subspecies until evidence to the contrary is presented.

#### Cheilosia caerulescens calculosa

Taxon classificationAnimaliaDipteraSyrphidae

Skufjin

Fig. 5

Cheilosia caerulescens calculosa Skufjin, 1977:57.

##### Type locality.

‘Lipetzk Region, Galichia Gora reserve’ (Russia).

##### Type material studied.

Holotype, ♂, in ZIN. The holotype is labelled: ‘Lipetzkaya obl. Galichia gora 18.V.1963 Skufjin’.

##### Description.

♂: Face in frontal view slightly divergent from level of antennal insertion to lower mouth edge, slightly elongated, shining with stripe of pollinosity. Facial knob moderate, shining (Fig. 5A). Parafacia shining with slight pollinosity, moderate in width, in broadest part about 2/3 of the width of basoflagellomere. Frons very slightly swollen, shining, with white and posteriorly some black pile. Frontal angle slightly < 90°. Lunula dark-brown. Antennal pits separated. Vertical triangle with black pile. Eye-contiguity approximately equal to length of frons without lunula. Basoflagellomere oval, yellow, arista brownish, almost bare, with very short pile (Fig. 5B). Scutum black, shining with fine punctuation, covered with dense erect bright yellow pile of equal length, with black pile of same length intermixed. Postalar callus with black bristles. Scutellum hind margin with some long fine yellow and black pile. Pleurae with rather dense gray pollinosity and yellow waving on the end pile. Dorsal and ventral hair patch of katepisternum connected. Femora black with yellow extreme tips; mesofemur with long yellow pile on posterior surface of basal 4/5 and black pile of apical 1/5. Pile on anterior surface of metafemur long and yellow in basal 2/3 and short and depressed in apical 1/3. Tibiae yellow with black ring, tarsi yellow with black fifth segment. Halter yellow. Wing completely covered with microtrichia, with brown spot on cross-veins. R_4 + 5_ of wing not distinctly curved. Abdomen oval, shining black covered with erect yellow pile. Sternite I and IV with pollinosity, sternites II–III shining. Genitalia as in Fig. 5E–G.

*Female*: Face as in male, parafacia shining, narrow, about 1/3 of width of basoflagellomere. Frons comparatively broad with two furrows (Fig. 5D). Vertical triangle with black pile. Basoflagellomere slightly broader than in male (Fig. 5C). Pilosity of scutum and scutellum as in male, but all bristles on scutellum hind margin yellow. Mesofemur with long yellow pile on posterior surface. Tibiae yellow with rather narrow black ring. Tarsi yellow with darkened 5th segment. Wing and abdomen as in male.

*Size*. Body length 9–10.2 mm.

##### Additional material studied.

**Russia** 4 ♂, 2 ♀ ‘Lipetzk Region, Galichia Gora reserve, 24–28.IV.1966, Kuznetzova’ [VSU]; 1 female ‘Lipetzk Region, Galichia Gora reserve, 12.5.1974, V. Kuznetsova’ [ZIN]; 1♂, 1 ♀ same place 24–25.IV.1966, (V. Kuznetzova) [ISEA].

##### Distribution.

European part of Russia.

##### Remark.

We treat the taxon as subspecies until evidence to the contrary is presented.

#### Cheilosia circassica sp. n.

Taxon classificationAnimaliaDipteraSyrphidae

http://zoobank.org/A9BCD04B-A7D9-48A5-A8C3-8B479858A16A

Fig. 6

##### Type material studied.

Holotype, ♂, pinned, with genitalia dissected in microvial, in ZIN. The holotype is labelled: ‘Teberdinskij zapov., okr. Usad’by, Kavkaz, Gorodkov, 9.VII.[1]968’, ‘g. Mal. Khatipara, verch. granitsa lesa, 2400 m’, ‘subalpinskij lug’ [Teberdinskii reserve, mountain Malaia Khatipara, close to border forest, 2400 m, subalpine meadow] [North Caucasus, Karastschaevo-Tscherkessia].

Paratype, 1 ♂, Teberdinskij reserve, mountain Malaia Khatipara, 1400–2000 m 14.VII.1982 (Lukashova) (ISEA).

##### Description.

♂: Face in frontal view slightly divergent from level of antennal insertion to lower mouth edge, with weak narrow strip of pollinosity (Fig. 6A). Facial knob rounded, not very protruding, shining. Parafacia pollinose in upper part and shining in lower part. Parafacia in its broadest part slightly more than 1/2 the width of basoflagellomere. Frons very slightly swollen, shining, with dense white wavy pile. Frontal angle ≈ 90°. Lunula brown. Antennal pits confluent. Vertical triangle with white pile. Eye-contiguity shorter than the length of frons without lunula. Basoflagellomere yellowish brown, rounded (Fig. 6B). Arista with short pile. Scutum, scutellum and pleura shining, with only very pale yellowish (almost whitish) pile, longer pile on pleura and posterior part of scutum distally wavy. Scutum and scutellum completely without black bristles or bristly like pile. Postalar callus without black bristles. Anepisternum and katepisternum shining, with white pile. Katepisternum with dorsal and ventral hair patches connected. Legs mainly black, knees narrowly yellow, tibiae also narrowly yellow at the extreme tip, 1–3 segments of meso and 2–3 segments of metatarsi yellowish. Apical half of posterior surface of mesofemur with long light pile. Metafemur with short and long white pile ventrally, apicoventrally with a few black pile. Halter yellow. Wing with all cross-veins darkened, completely microtrichose, R_4 + 5_ of wing not distinctly curved. Abdomen oval, shining, densely punctated. Pile erect and white, longer on the lateral parts and shorter on the central parts of tergites. Sternite I with pollinosity, sternites II–IV shining, pile erect, white. Hypopygium as on Fig. 6D–F.

*Size*. Body length 9 mm.

##### Etymology.

The specific name means “from Tscherkessia” in Latin and English.

##### Additional material studied.

No additional material available.

##### Distribution.

Russia (Caucasus).

##### Remark.

Female unknown.

#### Cheilosia herculana

Taxon classificationAnimaliaDipteraSyrphidae

Brădescu

Fig. 7

Cheilosia herculana Brădescu, 1982: 13.
Nigrocheilosia herculana : Vujić 1996: 56.

##### Type locality.

“Roumaine, Carpates Meridionales, Monts Mehedinti, Baile Herculane, Vallee du ruisseau Feregari” [Herculane, Romania].

##### Type material studied.

Holotype, male, in MGAB [not available for study]. Paratypes, a male and a female. Paratypes are labelled: ’Romania, Baile Herculane, 5.IX.1980, leg. VL Brădescu’, in coll. Claussen.

##### Description.

♂: Face in frontal view slightly divergent from level of antennal insertion to lower mouth edge, shining, with very weak stripe of pollinosity, lower part somewhat protruded (Fig. 7A). Parafacia rather narrow, approximately 1/2 the width of basoflagellomere, shining, with slight pollinosity on upper part. Frons slightly swollen, shining, with white and posteriorly some black, pile. Frontal angle ≈ 90°. Lunula black. Antennal pits confluent. Vertical triangle with black and a few yellow pile. Eye-contiguity slightly shorter than length of frons without lunula. Basoflagellomere small, pale-brown to dark-brown, arista rather long, with short pile (Fig. 7B). Scutum shining, rather coarsely punctated, all covered with whitish pile of even length, also on scutellum. Scutellum margin without stronger bristles. Anepisternum and katepisternum partly shining, with white long pile. Katepisternum with dorsal and ventral pile patches broadly divided posteriorly and narrowly connected anteriorly. Halter brownish. Wing with all cross-veins infuscated. Cell bm and cell cup of wing with obvious bare areas (Fig. 7H). R_4 + 5_ of wing not distinctly curved. Postalar callus without black bristles. Legs with distal part of femora and apical 1/3–1/2 and distal 1/6 of tibiae yellowish, tarsi yellow to yellowish brown, pile mainly white. Mesofemur posteriorly with long yellow pile. Metafemur ventrally with short black (rather strong) pile, distally dorsally with yellow pile, its anterior surface with long yellow pile. Abdomen shining, with pollinosity on medial anterior 2/3 of tergite II, and on medial anterior 1/3 of tergite III. Abdomen with white erect pile, longer on the sides and shorter medially. Hypopygium Fig. 7E–F.

♀: Face and parafacia as in the male. Frons shining, coarsely punctated, with white erect pile, and mixed with black erect pile on posterior part. Frontal furrows moderate (Fig. 7D). Lunula dark-brown. Basoflagellomere yellowish brown. Scutum shining, with white pile of even length. Otherwise as the male.

*Size*. Body length 10–11 mm.

##### Additional material studied.

No additional material available.

##### Distribution.

Romania, Montenegro, FYR Macedonia.

#### Cheilosia hercyniae

Taxon classificationAnimaliaDipteraSyrphidae

Loew

Fig. 8

Cheilosia hercyniae Loew, 1857:596; Marcos García 1985:518; Čepelák 1986: 53; Brădescu 1991: 39.
Chilosia hercyniae : Becker 1894:379; Sack 1932: 78; Šuster 1959:75; Séguy 1961:40.
Nigrocheilosia hercyniae : Vujić 1996:57.

##### Type locality.

“Der Hartz, Oesterreich” [sic][Austria].

##### Type material studied.

Lectotype, ♂, pinned, in MNB, here designated to fix the concept of *Cheilosia hercyniae* Loew and to ensure the universal and consistent interpretion of the same. The original description concerns only the male sex. The lectotype is labelled: ‘Austria, Schiner’, ‘Coll. H. Loew’, ‘Zool. Mus., Berlin’, ‘Lectotype *Cheilosia hercyniae* Loew, Ståhls & Barkalov des.’.

##### Description.

♂: Face in frontal view moderately divergent from level of antennal insertion to lower mouth edge, with slight to moderate pollinosity, but facial knob shining. Facial knob moderately protruding (Fig. 8A). Parafacia rather broad, approximately 2/3 of width of basoflagellomere, with slight pollinosity in upper part and shining in lower part and with short white pile. Frons moderately swollen, shining, with black pile. Frontal angle slightly > 90°. Lunula reddish brown. Antennal pits separated. Vertical triangle with black pile. Eye-contiguity nearly equal to the length of frons without lunula. First and second antennal segment dark-brown, third segment somewhat quadratic, bright orange with dorsal margin black (Figs 8B, 12C). Arista pubescent, with very short pile. Scutum shining (bluish ting), finely punctated, with whitish to yellowish erect, rather long, pile of about even length, intermixed with black pile of same length or slightly longer, also on scutellum, margin with 8–10 longer black bristles. Postalar callus with 3–5 bristles. Anepisternum and katepisternum shining, with slight pollinosity, and with whitish and black, rather long, pile intermixed. Katepisternum with dorsal and ventral hair patches broadly divided. Wing brownish. Wing completely microtrichose. R_4 + 5_ of wing not curved. Halter yellow, knob dark-brown. Legs with distal part of femora, tibia with basal 1/3–1/2 and distally narrowly yellow, tarsi dorsally dark-brown. Mesofemur posteriorly with long yellow pile and with some black ones on the tip; metafemur ventrally with short black (rather strong) pile, distally dorsally with longer black and yellow pile, its anterior surface with long yellow pile in basal 2/3 and with black pile in apical 1/3. Abdomen slightly oval, shining tergites I–III with brownish pollinosity medially, tergite IV with stripe of pollinosity antero-medially. Pile yellow, erect on all tergites, tergite IV on postero-lateral corners also with black pile. Pregenital segments with pollinosity, with yellow pile. Sternites pollinose with yellow erect pile, sternite IV also with short appressed black pile medially. Hypopygium as on Fig. 8E–F.

♀: Face and parafacia as in male. Basoflagellomere big, bright orange-yellow, quadratic, with a long (≈ 1/3 of the length of antenna) groove on the inner ventral side (Fig. 8C). Anterior 1/3 of frons very shiny, with fine punctation, and very short pile, posterior 2/3 shining, coarsely punctated, with short yellow and longer black erect pile (Fig. 8D). Scutum shining, rather coarsely punctated, with short erect yellow and slightly longer black pile. Scutellum margin with 4–6 rather strong black bristles. Legs as in the male. Wing yellowish. Abdomen oval, shining, densely punctated, tergite I and antero-medial part of tergite II with pollinosity. Pile erect, yellow on lateral parts, long on lateral part of tergite II, and short, black, appressed pile medially. Sternites with slight pollinosity. Otherwise as the male.

*Size*. Body length 8–11 mm.

##### Additional material studied.

**Italy** 1 ♂ Italy, Val Gardena, Col Raiser-n. Regensb. Hütte, 2150–2200 m, 46°35'N 11°44'E, 22.06.2013, leg. T. & W. Romig [coll. Romig]; **France** 1 ♂ ‘Le Lautaret, 10.VII.24’ [MNHN], 1 ♂ ‘Le Lautaret, 31.VII.26’ [MNHN], 1 ♂ ‘Le Lautaret, 5.VIII.23’ [MNHN], 1 ♂ ‘Le Lautaret, 4.VIII.25’ [MNHN]; **Montenegro** 1 ♂ Montenegro, Durmitor, Krecmani, 30.VII.1999, leg. Vujić [FSUNS]; **Switzerland** 1 ♂ ‘Helvetia, GR, Ftan /Clünas, 2 100 m, 5.VIII.1996, Merz & Bächli’ [ETH]. See also Table 2 for data molecular specimen vouchers also used for morphological study.

##### Distribution.

Austria, Czech Republic, France, Germany, Italy, Montenegro, Poland, Romania, Spain, Switzerland,

#### Cheilosia kerteszi

Taxon classificationAnimaliaDipteraSyrphidae

Szilády

Fig. 9

Chilosia kerteszi Szilády, 1938:138.
Cheilosia kerteszi : Brădescu 1991: 39.
Nigrocheilosia kerteszi : Vujić 1996:59.

##### Type locality.

“Ostungarn, Szászka und Rév” [Romania].

##### Type material studied.

Type material could not be located, apparently lost.

##### Description.

♂: Face in frontal view slightly divergent from level of antennal insertion to lower mouth edge, shining with very slight pollinosity, lower part somewhat protruded (Fig. 9A). Facial knob rounded, shining, moderately protruded. Parafacia rather narrow, in the upper part approximately 1/2 the width of basoflagellomere, in lower part slightly narrowed. Parafacia shining, or with slight pollinosity on upper part. Frons slightly swollen, shining, with predominantly yellow and a few black pile. Frontal angle ≈ 90°. Lunula black. Antennal pits separated. Vertical triangle with black and a few yellow pile. Eye-contiguity slightly shorter than length of frons without lunula. Basoflagellomere small, brownish, arista rather long, almost bare (or with very short pile) (Fig. 9B). Scutum shining, rather densely punctated, all covered with erect yellow and black pile of even length, pile becoming slightly longer towards scutellum. Postalar callus with few black bristles. Scutellum with only yellow pile, margin without longer black bristles. Anepisternum and katepisternum with slight pollinosity, with yellow long pile. Ventral and dorsal pile patches on katepisternum narrowly connected posteriorly. Halter yellow. Wing with all cross-veins infuscated, completely covered with microtrichia. R_4 + 5_ of wing not curved. Legs dark, with apical (1/8) part of femora, and basal 1/2 and apical 1/6 of tibiae yellowish, tarsi yellow, fifth tarsal segment dorsally blackish or brownish. Mesofemur posteriorly with white pile, metafemur anteroventrally and -dorsally with long white pile, and ventrally with shorter (stronger) black pile. Abdomen shining laterally, mid parts of tergites I–III with pollinosity. Tergites with yellow erect pile, longer on the sides and shorter medially. Sternite I pollinose, other sternites shining, pile yellow, erect. Hypopygium as on Fig. 9E–G.

♀: Face and parafacia as in the male. Frons coarsely punctated, with white erect pile, and also with black erect pile on posterior part (Fig. 9D). Parafacia < 1/2 the width of basoflagellomere. Basoflagellomere reddish (Fig. 9C). Postalar callus with no black bristles. Scutum shining, with yellow and more scattered black pile of even length. Abdomen shining, tergite I and anterio-medial part of tergite II with slight pollinosity. Otherwise as the ♂.

*Size*. Body length 10–11 mm.

##### Additional material studied.

**Serbia** 1 ♂, 1 ♀ ’Serbija, Klisura peka, 3.V.1993, leg. Milankov’ [FSUNS].

##### Distribution.

Montenegro, Romania, Serbia*.

#### Cheilosia laeviventris

Taxon classificationAnimaliaDipteraSyrphidae

Loew

Fig. 10

Cheilosia laeviventris Loew, 1857:602; Čepelák 1986:53; Brădescu 1991:38; Doczkal 1995:21; Barkalov and Ståhls 1997:35; Stuke 2000: 22.
Chilosia laeviventris : Becker 1894:358; Sack 1932:82; Šuster 1959:71.
Chilosia primulae Hering, 1944:117. Type locality: ‘Ebenstein, Hochschwabgruppe’ [Austria]. **Syn. n.**

##### Type locality.

“Oesterreich” [sic] [Austria].

##### Type material studied.

*C. laeviventris*: Lectotype, ♂, pinned, with genitalia dissected in microvial, in ZMHU, here designated to fix the concept of *Cheilosia laeviventris* Loew and to ensure the universal and consistent interpretation of the same. The original description is only for the male sex. The lectotype is labelled: ‘Austria, Schiner’ [handwritten, faded inc], ‘laeviventris’ [handwritten, faded inc], ‘coll. H. Loew’, ‘11469’, ‘*Cheilosia laeviventris* m.’ [handwritten, faded inc], ‘*Cheilosia laeviventris* Loew, Ståhls & Barkalov des.’.

*C. primulae*: Lectotype, ♂, ZMHU, here designated to fix the concept of *Cheilosia primulae* Hering and to ensure the universal and consistent interpretation of the same. The lectotype is labelled: ‘Ebenstein, Hochschwabgruppe, leg. H. Franz’, ‘*Cheilosia primulae* m. det. Hering 1944’ [handwritten, faded inc], ‘Zool. Mus. Berlin’, ‘Lectotype *Cheilosia primulae* Hering, Ståhls & Barkalov des.’. Genitalia dissected. A female paralectotype, idem. Both *C. primulae* are reared from larvae found in *Primula auricola* L. and the pupae are mounted on the same pin. The lectotype is forced out from the pupa: left wing missing, right wing incomplete, only with right hind leg, face slightly demolished.

*Description*. ♂: Face in frontal view moderately divergent from level of antennal insertion to lower mouth edge, with pollinosity, lower part of face clearly protruded (Fig. 10A). Parafacia pollinose, moderate in width, approximately 2/3 of width of basoflagellomere. Frons shining, with black pile. Frontal angle slightly > 90°. Lunula dark-brown to black. Antennal pits separated or very narrowly connected. Vertical triangle with black pile. Eye-contiguity nearly equal to or slightly shorter than length of frons without lunula. Basoflagellomere small, rounded, reddish brown to dark-brown (Fig. 10B). Arista moderately long, with short pilosity. Scutum and scutellum shining, densely punctated, with erect, mixed yellow and black pile, of about equal lengths. Central part of scutum with a rather broad transversal band of black pile. Hind margin of scutellum with black bristles or bristle-like pile. Postalar callus with some black bristles. Pleura slightly pollinose, pile predominantly black or mixed. Katepisternum with dorsal and ventral hair patches only narrowly connected anteriorly. Legs black, knees sometimes very narrowly yellowish. Apical half of posterior surface of mesofemur with long black pile, and basal part of anterior surface of metafemur with short black pile. Wing completely microtrichose, with all crossveins infuscated, R_4 + 5_ not distinctly curved (Fig. 10H). Abdomen shining with erect, yellow longer pile on lateral parts of tergites, and erect, black pile on central parts of tergites II–III, IV tergite with depressed pile. Tergite I–II with pollinosity medially on all length of tergite, tergite III with pollinosity only narrowly anteriorly or on all length of tergite. Tergite II often with a patch of black pile laterally. Sternites shining. Hypopygium as on Fig. 10E–G.

♀: Face as in the ♂. Frons with pollinosity laterally, otherwise shining, pile mixed black and white (Fig. 10D). Lunula brown. Basoflagellomere small, rounded, brown to dark-brown (Fig. 10C). Arista with short pile. Scutum shining, pile as in male. Otherwise as the ♂.

*Size*. Body length 8–11 mm.

##### Material studied.

**Austria** 1 ♂, 2 ♀ ‘Österreich, Tirol, Seefelder Joch, 2050 m, 15.VII.1969, v. d. Goot & Lucas’ [ZMA]; **Germany** 5 ♂ ‘Alpen Oberstdorf, Nebelhorn, Koblat, 1 920–2 220 m, 4.7.1994, D. Doczkal’ [coll. D. Doczkal].

##### Distribution.

Austria, France, Germany, Italy, Romania, Switzerland.

#### Cheilosia venosa

Taxon classificationAnimaliaDipteraSyrphidae

Loew

Fig. 11

Cheilosia venosa Loew, 1857:603; Barkalov and Ståhls 1997:63.
Chilosia venosa : Becker 1894:356; Sack 1932:106; Šuster 1959:70.

##### Type locality.

‘Oesterreich’ [sic] [Austria].

##### Type material studied.

Lectotype ♂, pinned, with genitalia dissected in microvial, in MNB, here designated to fix the concept of *Cheilosia venosa* Loew and to ensure the universal and consistent interpretion of the same. The original descriptions is only for the male sex. The lectotype is labelled: ‘Austria, Schiner’ [handwritten, faded inc], ‘Coll. H. Loew’, ‘Zool. Mus., Berlin’, ‘Lectotype *Cheilosia venosa* Loew, Ståhls & Barkalov des.’.

##### Description.

♂: Face in anterior view moderately divergent from level of antennal insertion to lower mouth edge, with lower part not very prominent, with band of pollinosity (Fig. 11A). Facial knob prominent (nose-like). Parafacia with pollinosity, on lower part shining patch, slightly more than 1/2 the width of basoflagellomere. Frons shining, rather swollen, with black and white pile. Frontal angle ≈ 90°. Lunula dark-brown to blackish. Antennal pits confluent (occasionally only narrowly confluent). Vertical triangle with black pile. Eye-contiguity nearly equal to length of frons without lunula. Basoflagellomere small, rounded, brown to reddish brown. Arista short, thickened, clearly pubescent (Fig. 11B).

Scutum densely punctated, shining, with long, erect yellow pile, medially a transverse band of predominantly black pile. Scutellum on hind margin without (occasionally with some) black bristles. Postalar callus with two bristles. Pleura pollinose, with long black and yellow pile. Dorsal and ventral pile patches of katepisternum only narrowly connected anteriorly. Legs black, knees sometimes yellow, tibia sometimes black ringed. Apical half of posterior surface of mesofemur and basal part of anterior surface of metafemur with long black pile (Fig. 12A). Halter yellow. Wing with all crossveins infuscated, R_4 + 5_ distinctly curved (Figs 11I, 12A). Wing completely microtrichose. Abdomen shining, with pollinosity on middle part of tergites I–III. Tergites with long, erect, yellow pile laterally, and black short erect pile medially. Sternites I pollinose, II–IV shining. Hypopygium as on Fig. 11F–H.

♀: Face shining with fine pollinosity on the sides. Parafacia comparatively narrow, pollinose. Frons moderately broad, shining (Fig. 11D), with yellow and black pile (Fig. 11E). Basoflagellomere, small, rounded, black to brownish, with sensory pit (Fig. 11 C). Arista short, with dense, short pile, almost on whole length of arista. Scutum shining, with long, erect yellow and black pile of equal length. Pleura slightly pollinose. Scutellum margin without black bristles. Otherwise as the ♂.

*Size*. Body length 9–11 mm.

**Additional material studied. Austria** 1 ♂ ‘Österreich, Tirol, Seefelder Joch, 2 050m, 15.VII.1969, v.d. Goot’ [in ZMA], 1 ♂ ‘Austria, Brauer’ [MNB], 1 ♂ ‘Austria, Schiner’ [MNB]; **Switzerland** 1 ♀ ‘Helvetia, GR, Lenzerheide, Parpaner Rothorn, 2 850 m, 14.VII.96, B. Merz’ [ETH]; **Italy** 1 ♀ ‘Südtirol, Val di Planol, 2 000–2 400 m, 7.VII.1988, C. Claussen’ [in MZH], 1 ♀ ‘Stelvio Pass, 1 600–2 000 m, 22.VII.1988, Daccordi’ [in MZH], 1 ♀ ‘Valle Aurina, Bolzano, Val d. Vento, 2 400 m, 11.8.1987, Daccordi’ [in MZH].

##### Distribution.

Austria, Italy*, Germany, Romania, Switzerland*.

## Molecular COI analyses

### Sequences

COI barcodes were obtained of 658 bp sequence for 27 *Cheilosia* specimens and two outgroups (see Table 2), including five barcodes for *Cheilosia caerulescens*. The pairwise uncorrected p-distances for *Cheilosia caerulescens* samples ranged between 0–1.3%. COI barcodes were also obtained for representatives of subgenus Taeniocheilosia, and a few samples representing Cheilosia s. str. and sg. Eucartosyrhus Barkalov (Table 2).

### Phylogenetic trees

**Figure 13.**
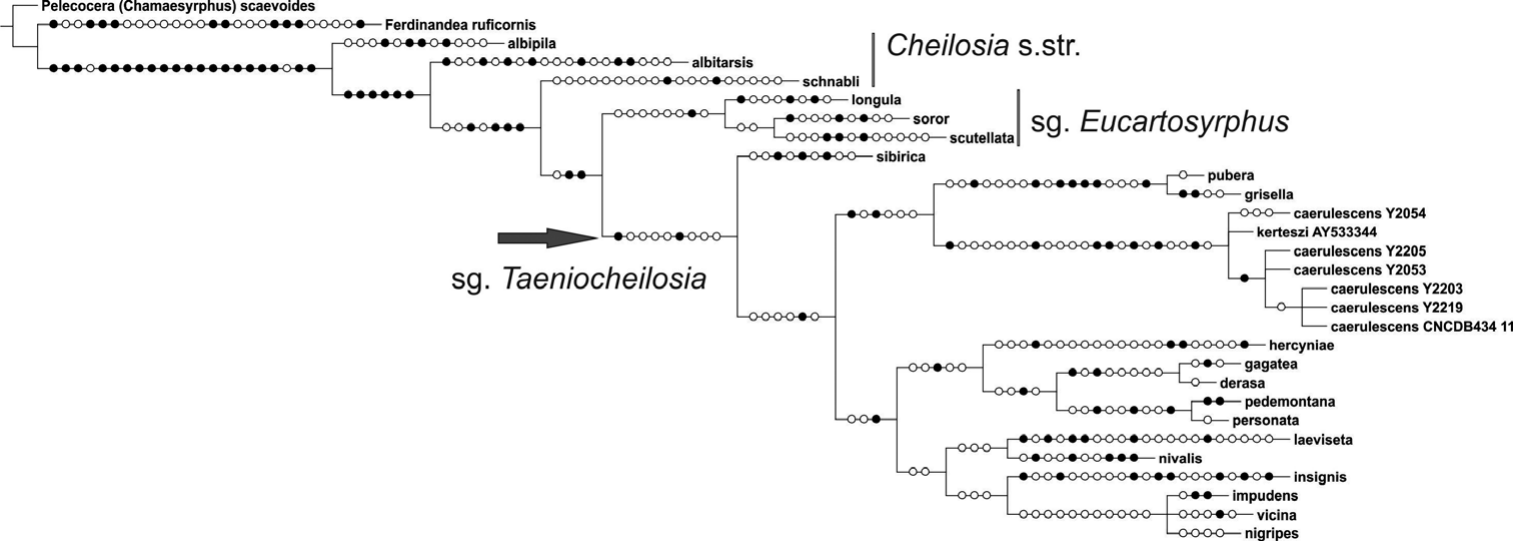
Parsimony analysis based on mtDNA COI barcodes. Strict consensus tree of two equally parsimonious trees. Nucleotide changes indicated, filled circles denote unique changes to this dataset, open circles non-unique.

**Figure 14.**
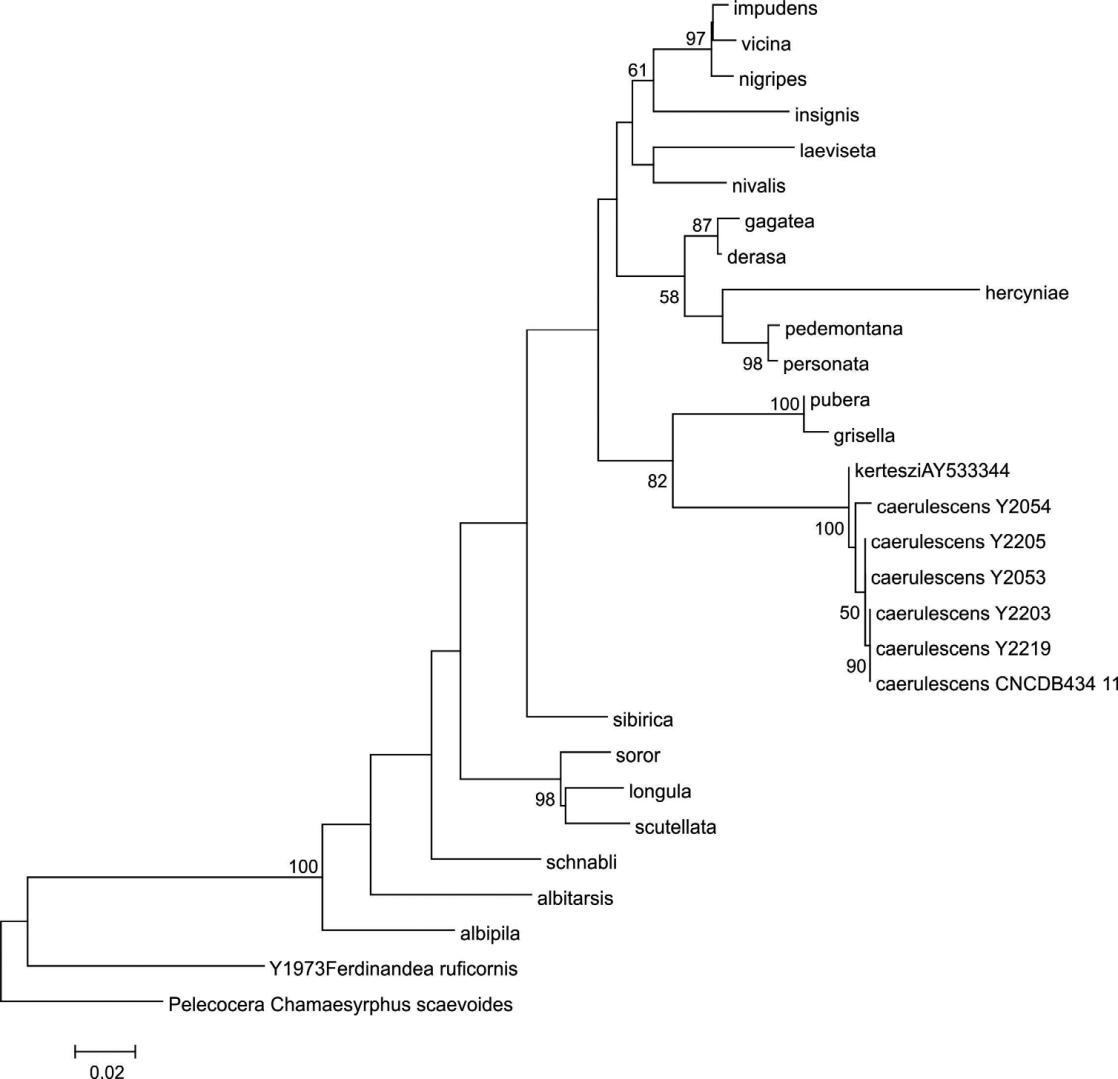
Maximum Likelihood tree with bootstrap support values >50% indicated above branches.

Parsimony analysis of the COI data resulted in two equally parsimonious trees of length 494 steps, with Consistency Index of 0.52 and Retention Index of 0.69, and the strict consensus is presented in Fig. 13. The parsimony analysis of the COI barcode data recovered the subgenus Taeniocheilosia as monophyletic with *Cheilosia sibirica* as sister group to the other included taxa. Cheilosia (T.) hercyniae was resolved among the other sg. Taeniocheilosia taxa. The obtained ML tree resolved the same topology the as the parsimony analysis (Fig. 14), except for slight change in position for *C. hercyniae*. In the parsimony tree Cheilosia (Taeniocheilosia) hercyniae was resolved as sister group to ((*C. gagatea* Loew, 1857+ *C. derasa* Rondani, 1857) + (*C. pedemontana* Rondani, 1857 + *C. personata* Loew, 1857)), and in the ML tree as sister group to (*C. pedemontana* + *C. personata*).

## Discussion

The Cheilosia (Taeniocheilosia) caerulescens species group presently comprises eight species. Their distribution is predominantly in the mountainous areas of Europe and Caucasus, but *C. caerulescens calculosa* is known from chalk slopes at the of the river Don in Lipetsk region (Russia) and *C. caerulescens caerulescens* is widely distributed in central Europe including both lowland and mountainous areas. The larval host plant is known for *C. caerulescens caerulescens*, the larvae are phytophagous mining the leaves of species of genus *Sempervivum* (Aguilar and Coutin 1988, Stuke 2000) and for *C. laeviventris* (as *C. primulae*) with larvae reared from *Primula auricola* (Hering 1944). The taxon has spread in last decades and was reported for the first time for Netherlands by van der Goot in 1989 and in Great Britain in 2008 by Collins and Halstead, and has been listed as a garden pest species in the Netherlands by Reemer (2003).

We consider the *C. caerulescens calculosa* subspecies as a relict form. According to Skufjin and Kuznetsova (1982) this subspecies “belongs to boreomontane relict forms associated with lower alpine plants growing on poor limestone and marl soils on steep slopes of Central Russian Upland”. The larval host plant is unknown for this taxon. In a forthcoming study we aim to resolve the rank of the taxon, specific or subspecific, by obtaining fresh samples of the taxon from the type locality for molecular analysis.

Neither the parsimony analysis nor the ML analysis resolved *Cheilosia hercyniae* among the included *C. caerulescens* group species, but among other sg. Taeniocheilosia taxa in congruence with conclusions based on morphological characters. *C. hercyniae* shares the character of infuscated wing cross-veins and bi-coloured legs with the members of the *Cheilosia caerulescens* group, but differs from all *C. caerulescens* group species in having a bright orange-yellow basoflagellomere and hair patches of katepisternum widely separated. In male genitalia *C. hercyniae* is similar to some sg. Taeniocheilosia species, e.g. *Cheilosia pedemontana*, *C. gagatea* and *C. faucis* (Becker, 1894) in the shape of the superior lobe.

The COI barcode of *Cheilosia kerteszi* is not full length and is very similar to the COI of the included *C. caerulescens* samples, but not identical to anyone of them. Identical or near identical COI barcodes of closely related Eristalinae species has also been recorded for *Merodon* species groups, see e.g. Šašić et al. (2016).

The studied group can be regarded as a morphologically intermediate group between the subgenera *Cheilosia* (*Neochilosia* Barkalov) and *Cheilosia* (*Eucartosyrphus* Barkalov), as the colour of the legs of its members is similar to that of the latter, but the presence of a large plate on the left process of superior lobe of hypandrium indicates an affiliation of this group to the subgenus Neochilosia.

## Supplementary Material

XML Treatment for Cheilosia armeniaca

XML Treatment for Cheilosia caerulescens caerulescens

XML Treatment for Cheilosia caerulescens calculosa

XML Treatment for Cheilosia circassica

XML Treatment for Cheilosia herculana

XML Treatment for Cheilosia hercyniae

XML Treatment for Cheilosia kerteszi

XML Treatment for Cheilosia laeviventris

XML Treatment for Cheilosia venosa
